# Comprehensive Metabolomics Profiling and Bioactivity Study of *Lycium shawii* (Awsaj) Extracts with Particular Emphasis on Potential Anti-Malarial Properties

**DOI:** 10.3390/metabo15020084

**Published:** 2025-02-01

**Authors:** Ruba Al-Nemi, Mutaz Akkawi, Khalid Sawalha, Siska Andrina Kusumastuti, Nuralih, Susi Kusumaningrum, Tia Okselni, Vania Chlarisa Situmorang, Abdi Wira Septama, Mariusz Jaremko, Abdul-Hamid Emwas

**Affiliations:** 1 Bioscience Program, Biological and Environmental Sciences and Engineering Division, King Abdullah University of Science and Technology (KAUST), Thuwal 23955-6900, Saudi Arabia; ruba.nami@kaust.edu.sa; 2 Life Sciences Department, Faculty of Science & Technology, Al-Quds University, Jerusalem P.O. Box 20002, Palestine; makkawi@staff.alquds.edu (M.A.); ksawalha@staff.alquds.edu (K.S.); 3 Research Center for Pharmaceutical Ingredients and Traditional Medicine, National Research and Innovation Agency, Cibinong, Kabupaten Bogor 16911, Indonesia; sisk001@brin.go.id (S.A.K.); nura008@brin.go.id (N.); susi004@brin.go.id (S.K.); tia.okselni@gmail.com (T.O.); vania.s@gmail.com (V.C.S.); abdi001@brin.go.id (A.W.S.); 4 KAUST Core Laboratories, King Abdullah University of Science and Technology, Thuwal 23955-6900, Saudi Arabia

**Keywords:** anti-malarial, bioactivities, *Lycium shawii*, mass spectrometry, NMR spectroscopy, untargeted metabolite profiling

## Abstract

**Background/Objectives:** Although malaria is one of the oldest known human diseases, it continues to be a major global health challenge. According to UNICEF, the global malaria mortality rate exceeded 600,000 annually in 2022, which includes more than 1000 children dying each day. This study aimed to investigate the comprehensive chemical profile and biological activities, particularly the antimalarial activity, of *Lycium shawii* (Awsaj), a shrub traditionally used in the Arabian Peninsula, Middle East, India, and Africa to treat a myriad of ailments. **Methods:** Crude extracts of *L. shawii* were prepared using water, ethanol, methanol, and acetone. Nuclear magnetic resonance (NMR) spectroscopy and mass spectrometry (MS) were utilized to perform untargeted metabolomics to maximize metabolite detection and tentatively identify bioactive phytochemicals. The total phenolic content (TPC) was measured for each extract, and bioassays were conducted to evaluate their antimalarial, antibacterial, and anti-inflammatory activities, particularly those of the water extract, which is the traditional method of consumption in Arabian folk medicine. **Results**: A total of 148 metabolites were detected, 45 of which were classified as phytochemicals. The bioassays revealed that the water extract that is traditionally used showed promising antimalarial potential by significantly inhibiting β-hematin formation in vitro at 1 mg/mL (with an absorbance of 0.140 ± 0.027). This is likely due to the rich presence of quinoline in the aqueous extract among several other bioactive phytochemicals, such as phenylpropanoids, alkaloids, flavonoids, and benzenoids. However, their anti-inflammatory and antibacterial activities were found to be weak, with only a minor inhibition of nitric oxide (NO) production in LPS-induced RAW 264.7 cells at a concentration of 500 µg/mL and weak antibacterial effects against pathogens like *P. aeruginosa*, *MRSA*, *A. baumannii*, and *K. pneumoniae* with an MIC of 500 μg/mL. The results also revealed that the methanolic extract had the highest TPC at 26.265 ± 0.005 mg GAE/g. **Conclusions:** The findings support the traditional medicinal use of *L. shawii* and highlight its potential as a source of novel therapeutic compounds, particularly for treating malaria. This study encourages further research to isolate and develop effective plant-based anti-malarial agents.

## 1. Introduction

Malaria is an ancient disease caused by *P. malariae*, *P. ovale*, *P. falciparum*, *P. knowlesi*, and *P. vivax* that still represents a serious global health challenge, particularly affecting populations in humid tropical regions where the disease is endemic [1]. According to UNICEF, every minute in Africa, a child under the age of five loses their life to malaria, and many of these tragic deaths could have been prevented or treated [2]. Plants have played a central role in traditional treatments for malaria across various cultures, containing a myriad of bioactive phytochemicals that are currently being utilized in pharmaceutical research, namely terpenes, alkaloids, polyphenols, and flavonoids, offering hope for more sustainable treatments, especially as drug resistance continues to pose challenges in existing malaria treatments [3,4,5].

While the concept of phytochemicals belongs to modern botany and biochemistry, the medicinal use of plants is ancient and spans the globe, with the ‘modern’ antimalarial drugs quinine and artemisinin providing one of the early examples of the transition from ancient, plant-based traditional medicine to modern ethno-pharmacology and biochemistry [6]. Both quinine and artemisinin have in turn led to the development of synthetic and semi-synthetic analogues (e.g., chloroquine, artesunate, etc.) and are now routinely used for the treatment of malaria, namely in the form of, e.g., chloroquine- or artemisinin-based combination therapies (ACTs) [7].

The starting point of the research reported in the present paper is similarly rooted in traditional medicine. *Lycium shawii,* a halophyte plant of the Solanaceae family, locally known as Awsaj in the Middle East and North Africa region, is a perennial, drought-resistant desert plant that has been used for centuries in herbal medicine as a dry powder and decoction to treat various conditions (Table 1) [8,9]. Some of these uses are supported by the ethnobotanical and pharmaceutical literature, in which it is reported that different parts of *L. shawii* possess a variety of bioactivities, such as antimicrobial [10,11], antidiabetic [12], antioxidant [10,13,14], anti-inflammatory [15], anti-plasmodial [16], and anticancer properties [14,17,18], but limited research exists on its anti-malarial properties.

Therefore, here, we focus on the potential antimalarial properties of *L. shawii*, building both on its traditional uses and modern biochemistry. Furthermore, examining multiple therapeutic properties in medicinal plants offers a holistic approach to addressing symptoms and concurrent infections. For instance, bacterial invasions often trigger immune responses that exacerbate inflammation, a process further intensified in malaria due to the breakdown of red blood cells and the release of toxic heme. Therefore, evaluating *L. shawii* for its anti-malarial, anti-bacterial, and anti-inflammatory potential could offer a comprehensive approach to treating malaria, symptoms, and concurrent infections.

Previous studies on *L. shawii* reveal its diverse phytochemical composition. For instance, Ur Rehman et. al. conducted a phytochemical analysis for the identification of diverse compounds in the stem bark of *L. shawii* collected from Oman using nuclear magnetic resonance (NMR) spectroscopy. The results from this study confirmed the presence of 14 natural compounds belonging to several bioactive classes, such as sesquiterpene lactones, triterpenoids, and steroids [20]. In another study, a quantitative analysis was carried out on the chemical composition of dried berries from *L. shawii* collected in Tunisia, leading to the identification of bioactive phenols and flavonoids [13]. In a more detailed study, leaf extracts of *L. shawii* collected in Egypt revealed the presence of diverse primary and secondary metabolites. In that study, the chromatographic analysis identified nine biologically active compounds using ultraviolet-visible absorption spectroscopy, ^1^H-NMR, ^13^C-NMR spectral data, and mass spectrometry. The separated compounds were identified as 2,3-dihydroxy benzoic acid, quercetin, gallic acid, rutin, p-coumaric acid, ferulic acid, quercetin 3-methoxy glucoside, quercetin 3, 7 diglucoside, and quercetin 3-O-β- glucoside [21].

Metabolomics involves the study of small molecules, known as metabolites, present within all living organisms and biological systems and which may be extracted from samples of plants, animals, and humans alike [22,23,24,25]. Metabolomics analyses can be classified into two main types: targeted and untargeted analyses. Targeted analysis focuses on quantifying a small number of metabolites, whereas untargeted analysis is used to profile and detect the maximum number of metabolites in the samples being studied [26]. Metabolomics is a powerful approach that utilizes a diverse range of analytical techniques, such as NMR spectroscopy and mass spectrometry (MS), along with gas chromatography (GC-MS) and liquid chromatography (LC-MS) [27]. Each analytical method presents distinct advantages and limitations that can significantly influence research findings. For instance, NMR spectroscopy is recognized as a non-destructive and unbiased technique characterized by its exceptional reproducibility and minimal sample preparation requirements [28]. However, NMR spectroscopy exhibits relatively low levels of sensitivity, and the potential for signal overlap may complicate data interpretation. In contrast, GC-MS and LC-MS are both highly sensitive and selective techniques that surpass NMR spectroscopy in terms of sensitivity [29]. Each method possesses unique strengths based on the specific metabolites under analysis. GC-MS is particularly effective for the examination of volatile compounds or those that may be rendered volatile through derivatization, making it suitable for volatile organic metabolites [30]. Conversely, LC-MS is better suited for the analysis of polar, thermally labile compounds that do not readily vaporize. Therefore, combining the application of these advanced techniques enables researchers to gain profound insights into metabolic processes, maximizing the detected and identified metabolites.

Thus, in our study, we applied an untargeted metabolomics approach using NMR spectroscopy fingerprinting alongside ultra high-performance liquid chromatography–electrospray ionization-mass spectrometry (UHPLC-ESI-MS) in positive and negative ionization modes and GC-MS analyses. Crude extracts were prepared with various solvents, namely water, methanol, ethanol, and acetone, to maximize metabolite detection and achieve a more comprehensive metabolomic profile. Additionally, we estimated the total phenolic content for each extract and investigated the antimalarial, anti-inflammatory, and antibacterial properties of the water extract, as it represents the traditional preparation method for *L. shawii* in Arabian folk medicine.

Our results reveal promising potential anti-malaria activity, demonstrated by the significant inhibition of toxic β-hematin formation, which is possibly linked to the abundant presence of quinoline in the aqueous extract of *L. shawii* and other phytochemicals with reported antimalarial activity.

## 2. Materials and Methods

### 2.1. Botanical Description and Collection

*Lycium shawii* is adapted to semi-desert and extreme desert climates and is predominantly native to Middle Eastern countries such as Palestine, Jordan, Iraq, Iran, Egypt, and Saudi Arabia, as well as other countries in the Indian subcontinent and some parts of Africa. It is a perennial shrub with many woody branches held erect from the ground, with long and sharp spikey thorns on the stem. Its leaves grow in clusters with white to pale violet flowers [19]. *Lycium shawii* is identified as a threatened species according to the red list of the IUCN.

**Figure 1:**
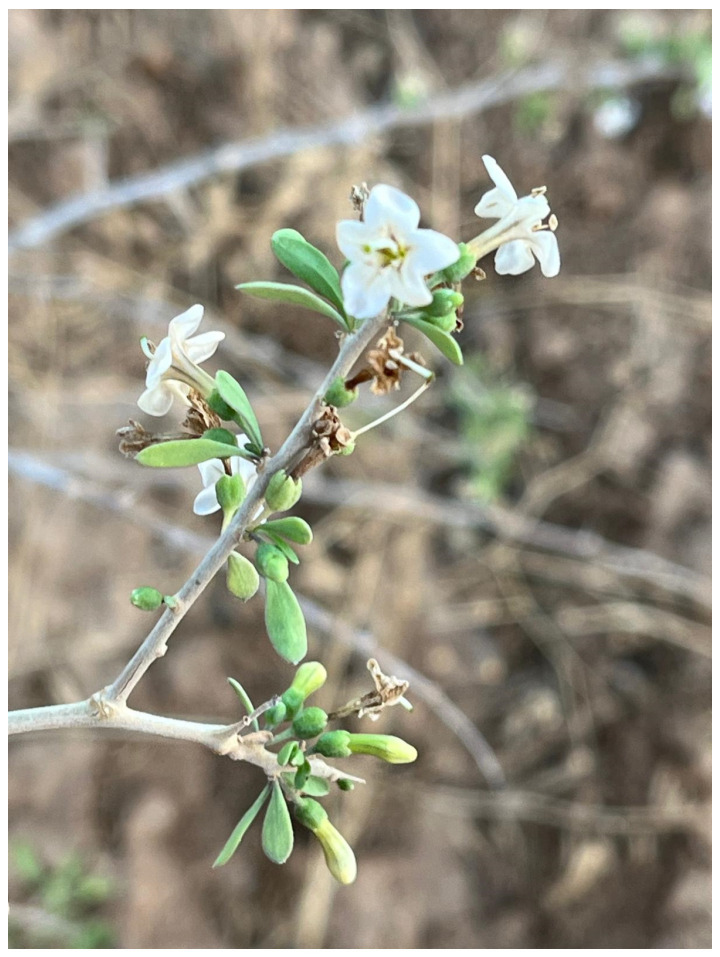
In situ photo of *Lycium shawii* samples. Photo by Khalid Sawalha in Masafer of Bani-Naim–Um Loqsub–Palestine.

The botanical material was identified and collected by Khalid Sawalha from a natural habitat on the eastern slopes of Bani-Naim, east of Hebron–West Bank–Palestine during the spring season of 2021 (Figure 1), catalogued in the herbarium of Al-Quds University, Palestine with voucher number (LS AQUBH21).

### 2.2. Extraction of L. shawii Samples

The plant leaves were air-dried in the shade at room temperature until constant weight was reached and then powdered using an electric grinder. To prepare crude extracts for the metabolomics analysis, 200 mg of the dry powder leaves was dissolved in each of the following solvents: 1.5 mL of Mili-Q water to obtain *L. shawii* water extracts (LSW), ethanol ≥99.8% (Honeywell Riedel-de Haen, Seelze, Germany) to obtain *L. shawii* ethanolic extracts (LSE), methanol ≥99.8% to obtain *L. shawii* methanolic extracts (LSM), and acetone ≥99.8% to obtain *L. shawii* acetone extracts (LSA) (VWR Chemicals, Radnor, PA, USA). The mixtures were placed on a shaker at room temperature for 15 h, vortexed, and centrifuged at 14,000× *g* for 2 min. Supernatants were transferred to 2 mL tubes, and an additional 0.5 mL of each solvent was added to the remaining residues, respectively, and extracted again at room temperature for 30 min. The supernatants were combined to obtain a total volume of 2 mL of crude extracts and stored at 4 °C prior to analysis.

To conduct the bioassays, crude extracts that reflect the traditional method of *L. shawii* consumption in Arabic folk medicine were prepared. Two grams of different parts of the *L. shawii* samples (leaves, stems, and seeds) were separately soaked in 150 mL of distilled hot water at 90 °C for 20 min. The mixtures were then incubated at room temperature, before being filtered through MN 615,110 mm filter paper. Dried and concentrated extracts from the infusions were obtained by evaporation of the water at 60–70 °C under reduced pressure using a rotary evaporator (IKA WEREKRV06-ML), followed by lyophilization (Labconco, Kansas City, MO, USA) until constant weight was achieved. The final dried extracts were stored in opaque bottles and kept in desiccators until analysis.

### 2.3. Metabolomic Profiling

#### 2.3.1. NMR Spectroscopy Analyses of *L. shawii* Extracts

Aliquots of each extract (500 µL) were dried in speed vacuum (Labconco, Kansas City MO, USA) then diluted in 600 μL of deuterium oxide (D 99.90%) for LSW, CD_3_OD (D 99.8%) for LSM, ethanol-D6 (D 99%) for LSE, acetone-D6 (D 99.9%) for LSA (all from Cambridge Isotope Laboratories, Inc., Tewksbury, MA, USA). The prepared samples were then transferred into 5 mm NMR spectroscopy tubes. The deuterated solvents contained tetramethylsilane (TMS) as an internal calibration standard (0.03~0.05%) at chemical shift (δ) of 0.0 ppm.

To obtain comparable data, all spectra were recorded under the same parameters and conditions on Bruker 800 MHz AVANCE NEO NMR spectrometer equipped with a TCI Bruker CryoProbe (BrukerBioSpin, Rheinstetten, Germany). For samples dissolved in D_2_O, the 1D ^1^H NMR spectra were recorded using the standard Bruker excitation sculpting water suppression pulse sequence (zgesgp), while the standard Bruker 90-degree excitation pulse sequence was used for the organic solvent extracts (zg). Bruker Topspin 4.0.7 software (Bruker BioSpin, Rheinstetten, Germany) was used for data collection using the following parameters: digital resolution of 32 K for complex data points; spectral width = 20 ppm; pulse duration = 8 μs; pulse power level = 9.256 W; relaxation delay = 5 s; acquisition time = 2 s; and number of scans = 128 using LB = 1 Hz prior to the Fourier transformation.

#### 2.3.2. GC-MS Analysis of *L. shawii* Extracts

To prepare for GC-MS analysis, 100 µL of each *L. shawii* extract and 200 µL of a certified 17-amino-acid standard mix solution (AA mix) (TraceCERT, Merck, Darmstadt, Germany) for quality control purposes were completely dried in a speed vacuum. For derivatization, 30 µL of MOX™ reagent (Thermo Fisher Scientific, Rockford, IL, USA) was added to the dried extracts and the AA mix, then incubated at 37 °C under continuous shaking for 90 min. Next, 50 µL of an MSTFA solution (Thermo Fisher Scientific, Bellefonte, PA, USA) was spiked with a C7-C40 n-alkanes standard mixture (10 µg/mL) (Dr. Ehrenstorfer, Augsburg, Germany) to determine the Kovats retention indices and was added to the samples and AA mix, then incubated at 37 °C for 30 min. The samples and AA mix were then centrifuged for 5 min, and 50 µL of each sample and AA mix was transferred to MS vials. For the pool sample, 10 µL of each replicate was transferred to a vial.

A Thermo Scientific Orbitrap Exploris GC 240 mass spectrometer (Waltham, MA, USA) operated in electron ionization (EI) mode was used for the analysis of the primary metabolites. Sample introduction was performed using a Thermo Scientific™ TriPlus™ RSH auto-sampler, and chromatographic separation of the volatile and semi-volatile components was achieved using a Thermo Scientific TRACE™ 1310 gas chromatograph (Waltham, MA, USA) equipped with Thermo Scientific Trace GOLD™ TG-5SilMS 30 m × 0.25 mm i.d. × 0.25 μm column (Waltham, MA, USA). The Orbitrap Exploris GC was tuned and calibrated using PFTBA to achieve mass accuracy of <1.0 ppm. The EI source was operated at 70 eV with an emission current of 50 µA. The temperature was set at 300 °C for the source and 150 °C for the mass analyzer. The scan range was 35–750 *m*/*z* with an orbitrap resolution of 30,000. The AGC target was set to standard, and the maximum injection time was set to auto mode. The data acquisition was lock-mass-corrected using GC column bleed siloxane of 207.03235 *m*/*z*. The GC inlet and transfer line temperatures were set at 250 °C and 280 °C, respectively, using helium as the carrier gas at a flow rate of 1.2 mL/min. The Trace 1310 GC was operated with an injection volume of 1 µL, and the sample was injected using a 10 µL syringe (in splitless mode) into a single gooseneck with glass wool Thermo Scientific™ LinerGOLD liner. The oven temperature was initially set at 70 °C for 2 min, then increased to 325 °C in increments of 10 °C/min and held at 325 °C for 8.5 min, with a total analysis time of 36 min.

#### 2.3.3. UHPLC-MS Analysis of *L. shawii* Extracts

For UHPLC-MS analysis, samples were prepared by speed vacuuming 100 µL of each extract until they were dry and reconstituting them in 150 µL of 80% acetonitrile. Then, they were vortexed and centrifuged for 2 min at 15,000 rpm before transfer of 130 µL of each sample into MS vials with glass inserts. For the pool sample, 10 µL of each sample was transferred to a vial. The measurement was performed using a Vanquish UHPLC System for rapid separation liquid chromatography coupled with IDX-Orbitrap HRMS (Thermo Fisher Scientific, Waltham, MA, USA). The samples were automatically infused (5 µL each) through the UHPLC system using the C18 column (Acquity CSH 2.1 × 100 mm, 1.7 µm) for the separation with a flow rate of 0.4 mL/min. The column was maintained at a temperature of 35 °C. The mobile phase was composed of solvent A (water) and solvent B (acetonitrile) *v*/*v*, both containing 0.1% formic acid. The gradient was linearly changed as follows: 0–1 min, 1% B; 1–10 min, 99% B; kept constant for 10–12 min; 12–12.01 min, 1% B; and 12.01–16 min, 99% B for column re-equilibration. The solvent for needle wash was in methanol/water (50:50, *v*/*v*). The Orbitrap ID-X mass spectrometer was calibrated using a commercially available Pierce™ FlexMix™ Calibration Solution (Thermo Scientific, Waltham, MA, USA) following the manufacturer’s guidelines. The IDX-Orbitrap HRMS was equipped with heated electrospray ionization (HESI) source using the following source parameters: sheath gas at 50 arb, auxiliary gas at 10 arb, spray voltage at 3500 V in positive mode and 2500 V in negative mode, capillary temperature at 300 °C, auxiliary gas heater temperature at 350 °C, and s-lens RF level at 45%. Full scan parameters were as follows: m/z range from 100 to 1000; mass resolution, 12,000; AGC target, 3e6; and maximum IT, 50 ms. DDA parameters in negative mode (ESI−) were as follows: m/z range from 100 to 1000, cycle time 0.7 s; intensity threshold to trigger MS/MS acquisition, 2 × 10^4^ dynamic exclusion, 6 s; assisted MSMS CE at 10, 30, 50, 70, and 90 (%); mass resolution of 60,000; AGC target, 1 × 10^5^; maximum IT, 118 ms; and *m*/*z* isolation window within 1.2. DDA parameters in positive mode (ESI+) were as follows: *m*/*z* range from 80 to 800, cycle time 0.7 s; intensity threshold to trigger MS/MS acquisition, 2 × 10^4^; dynamic exclusion, 6 s; assisted MSMS CE at 10, 30, 50, 70, and 90 (%); mass resolution, 60,000; AGC target, 1 × 10^5^; maximum IT, 118 ms; and *m*/*z* isolation window within 1.2.

### 2.4. Biological Activity Assays

#### 2.4.1. Determination of Total Phenolic Content (TPC) of *L. shawii* Extracts

The total phenolic content of the *L. shawii* extracts was determined using a modified Folin–Ciocalteu assay for 96-well microplates [31]. Various concentrations of gallic acid solutions were prepared in methanol (20, 40, 60, 80, 100, and 120 µg/mL) and used as standard to prepare a calibration curve. In brief, 20 µL of the extracts or gallic acid was added to 100 µL of aqueous solution of 10% Folin–Ciocalteu reagent and incubated for 10 min in the dark at RT, after which 100 µL of 7.5% Na_2_CO_3_ aqueous solution was added. The plates were then shaken for 10 s and incubated in the dark at RT for 30 min to allow the blue color to develop, indicating the oxidation of phenols in the extracts by the reagent. The assay was carried out in triplicate, and the absorbance was measured at 750 nm using a microplate reader (Cytation 5 Imaging Reader; Bio-Tek Instruments Inc., Winooski, VT, USA). The results are expressed as mg GAE/g of extract, calculated using the linear regression equation derived from the standard curve y = 0.0042x + 0.0682 (R2 = 0.9956). Data are expressed as the mean of three determinations ± standard deviation (SD).

#### 2.4.2. Cell Line Cultures

The RAW 264.7 macrophage-like cell line was obtained from the LAPTIAB-BRIN collections and cultured in DMEM medium supplemented with 10% FBS and 1% penicillin-streptomycin (100 units of penicillin/mL and 100 pg streptomycin/mL). Media and supplements were obtained from Gibco, Waltham, MA, USA. The cells were incubated at 37 °C with 5% CO_2_. The growth medium was replaced every 48 h.

#### 2.4.3. Viability Assay *L. shawii* Water Extract

The cytotoxicity assessment was performed using an MTT assay based on previous research [32], with slight modification. The RAW 264.7 cells were seeded in 96-well plates at a density of 2.0 × 10^4^ cells followed by 24 h incubation and then treated with 11 ranges of LSW extract concentrations (0.977–1000 µg/mL) for another 24 h. Viability or cytotoxic assay on RAW 264.7 was carried out to determine the non-toxic concentration of samples in RAW 264.7 cells to establish sample concentration in the nitric oxide (NO) inhibition assay. The medium was discharged, and the cells were washed using phosphate buffer saline (PBS) 1x. After adding 100 µL of a 0.5 mg/mL solution of 3-[4,5-dimethylthiazol-2-yl]-2,5 diphenyl tetrazolium bromide (MTT) into each well, the cells were incubated in incubator with 5% CO_2_ 37 °C for 4 h. To quantify the percentage of viable cells, the cells were exposed to a 100 µL solution containing 10% sodium dodecyl sulphate (SDS) in 0.1 N HCl overnight. The absorbance of the medium was measured at 570 nm using a microplate reader (Synergy HTX; Bio-Tek Instruments Inc., Winooski, VT, USA). Finally, the percentage of cell viability was calculated using the following formula: cell viability (%) = ([Abs of sample/Abs of control] × 100). A graph of cell viability percentage versus log concentration was plotted to determine the IC_50_ (concentration of the tested sample to reduce cell viability by 50%).

#### 2.4.4. In Vitro Semi-Quantitative Antimalarial Activity Assay of *L. shawii* Water Extract

Following the protocol by Deharo et al. [33], a semi-quantitative screening test for antimalarial activity was conducted. Dimethyl sulfoxide (DMSO) of 99.5% purity, chloroquine diphosphate salt, sodium acetate (99% purity), and hemin chloride were all purchased from Sigma-Aldrich, while glacial acetic acid was procured from Honeywell Fluka.

In this procedure, 50 μL of freshly dissolved hemin chloride (0.5 mg/mL) in DMSO and 100 μL of sodium acetate buffer (0.5 M, 4.4 pH) were combined with 50 μL of the LSW extract or the control (chloroquine) in a normal non-sterile flat-bottomed 96-well microplate and left for 18–24 h at 37 °C. The order in which the solutions were added was crucial for accurate results.

The plate was then centrifuged for 10 min at 2147 g. The supernatant was removed, and the pH of the reaction was measured. The final pH of the mixture should be between 5.0 and 5.2. The wells were washed with 200 μL DMSO per well to remove free hemin chloride. The plate was centrifuged again, and the supernatant was discarded. The remaining β-hematin was then dissolved in 200 μL of 0.1 M NaOH to form alkaline hematin, which can be measured spectrophotometrically. Finally, the absorbance was determined at 405 nm using an ELISA plate reader (Stat Fax-2100, Awareness Technology, Palm City, FL, USA). Ultrapure water was used as a negative control, whereas chloroquine dissolved in ultrapure water was used as a positive control.

#### 2.4.5. Anti-Inflammatory Activity Assay of *L. shawii* Water Extract

Anti-inflammatory activity was assessed using the inhibition of nitric oxide (NO) assay on RAW 264.7 cells following protocol [34]. NO production was determined using Griess reagent, and NO concentration in each group was quantified using sodium nitrite as a standard. RAW 264.7 cells were seeded into 96-well plates at a density of 10 × 10^5^ and incubated in 100 µL culture medium for 24 h. LSW extract of 12 different concentrations (0.24–500 µg/mL) were added into the cells and incubated for 2 h followed by the addition of 1 µg/mL LPS (lipopolysaccharide) into the RAW 264.7 cells. The cells were then incubated for another 24 h, after which 100 µL Griess reagent (0.1% N-(1-naphthyl)- ethylenediamine and 1% sulfanilamide in 5% phosphoric acid) was added. The mixture was incubated at room temperature for 20 min and then absorbance was measured using a microplate reader (Synergy HTX; Bio-Tek Instruments Inc., Winooski, VT, USA) at wavelength of 540 nm. To show that the sample has anti-inflammatory activity, the NO concentration of cells in the treatment group must be significantly lower than the NO concentration in the LPS-induced cells without treatment.

#### 2.4.6. Antibacterial Activity Assay of *L. shawii* Water Extract

A slightly modified broth microdilution method [35] was used to evaluate the antibacterial activity of LSW against selected clinical isolates of methicillin-resistant *Klebsiella pneumoniae, Pseudomonas aeruginosa*, and *Acinetobacter baumanii*. All experiments were conducted in a biosafety cabinet level 2, while adhering to proper safety precautions, such as those regarding hygiene, contact, environmental cleaning, isolation protocols, antimicrobial stewardship, and surveillance. All examined bacteria were cultured for 24 h at 37 °C on Brain Heart Infusion (BHI) agar. A few bacterial colonies were mixed with normal saline solution (NaCl 0.85%) to create the inoculate, and then the suspension’s turbidity was adjusted to match the standard 0.5 McFarland solution, which was stated to be equal to 1 × 10^8^ CFU/mL. To obtain a suspension with about 1 × 106 CFU/mL, it was diluted (1:100) with sterile normal saline solution. Next, to obtain the desired final concentration, each sample was first dissolved in DMSO and then diluted again, this time with BHI broth. A 96-well plate was used to prepare two-fold dilutions. After being introduced to each well, the bacterial suspensions (1 × 10^6^ CFU/mL) were incubated for 24 h at 37 °C. The lowest sample concentration that inhibited bacterial growth was determined to be the minimum inhibitory concentration (MIC) value.

#### 2.4.7. Data Processing and Statistical Analysis

Bruker Topspin 4.0.7 software was used to process and analyze the NMR spectra. After adjusting calibration, the phase of each NMR spectrum was corrected manually, and the baseline was adjusted automatically using the “abs n” command before obtaining the integration values. The chemical shift ratio (%) was calculated as the mean of three replicates ± SD, based on a previous study [36]. The AcquireX Deep Scan program (Thermo Fisher Scientific) was used on the pooled sample as an intelligent data-dependent Automated MSN data acquisition tool to obtain all precursor ions. Automated exclusion of background noise was performed, and inclusion lists were generated. The MS/MS data were automatically acquired on the pooled sample for identification of only the comprehensive fragmentation of relevant compounds in the samples. XCalibur software version 4.3 was used for GC-MS data acquisition. FreeStyle 1.8 SP2 was used to obtain total ion chromatograms, and Compound Discoverer version 3.3.2.31 (both from Thermo Fisher Scientific) was used to treat and process the UHPLC-MS and GC-MS data. For UHPLC-MS data processing, differences between the samples were identified, then retention time alignment, unknown compound detection, and compound grouping analyses were performed across all samples. Prediction of elemental compositions of all compounds was conducted, gap filling was applied across all samples, and chemical background noise (using blank samples) was hidden. Furthermore, mzCloud (ddMS2) and ChemSpider (formula or exact mass) were used to identify compounds. Similarity searches for all compounds were performed on ddMS2 data also using mzCloud, selecting only features with mzCloud Best Match score of 50>, annotation DeltaMass between −5.00 and 5.00 ppm, *p*-value < 0.05, and MS2 data. Spectra were manually compared to online values to confirm match. QC-based batch normalization was applied. Differential analysis was then calculated, before determining the *p*-values, adjusted *p*-values, ratios, fold change, and CV. For GC-MS data processing, gap filling (minimum value input) was used to replace zero values in compound grouping and statistical analysis, differential analysis was performed, Kovats retention indices were calculated, and unknown compounds were identified through NIST 14 library. Only annotations with calculated retention indices (RIs), RI delta < 200, and similarity indices (SI) >600 were selected. Spectra were manually compared to online databases to confirm match.

Lists of tentatively identified compounds were analyzed using the open-source Venn diagram tool (https://bioinformatics.psb.ugent.be/webtools/Venn/, accessed on 17 July 2024). Analyses were performed by MetaboAnalyst 5.0 (https://www.metaboanalyst.ca, accessed on 19 August 2024) using median-normalized, log-10-transformed, Pareto-scaled data. Further analysis of variance (ANOVA)-based heatmaps were created using the variable areas of the tentatively identified peaks with the Euclidean distance and Ward clustering method. Tentatively identified metabolite names were converted to the RefMet nomenclature (The Metabolomics Workbench) and classified using the online tool (https://www.metabolomicsworkbench.org/databases/refmet/name_to_refmet_form.php, accessed on 3 April 2024). Microsoft Office Excel (Microsoft Office 365) was used for statistical analysis and visualization. To calculate the percentages of cell viability, linear regression analysis was used to determine the IC_50_. The statistical mean difference among the samples was determined by ANOVA and Tukey post-hoc test. Pearson’s linear correlation and regression analysis was used to determine the association between variables. A *p* value of less than 0.05 was considered statistically significant.

## 3. Results and Discussion

### 3.1. Metabolomic and Phytochemical Composition of L. shawii Extracts

To enhance the detection and identification of metabolites, we used NMR spectroscopy, GC-MS, and UHPLC-MS in both positive and negative ionization modes. Additionally, we employed a variety of polar solvents including acetone, methanol, ethanol, and water to extract metabolites with the maximum level of diversity. Particular attention was given to bioactive phytochemicals such as polyphenols, flavonoids, terpenoids, and alkaloids.

**Figure 2:**
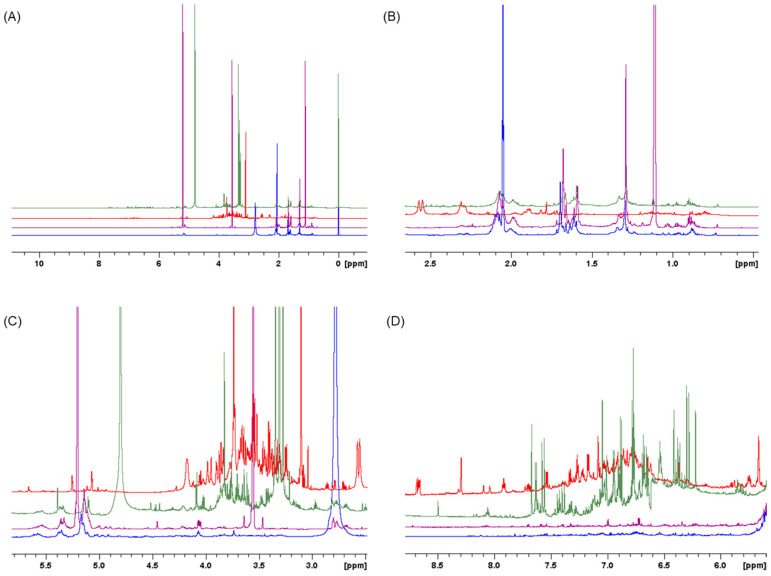
(**A**) Stacked ^1^H NMR spectra of *L. shawii* extracts from δ 0 to 10. Extended view of chemical shift regions is provided from (**B**) δ 0.5 to 2.7; (**C**) δ 2.5 to 5.5; and (**D**) δ 5.5 to 8.6. Spectra were acquired at 800 MHz. Blue: acetone extract; red: water extract; green: methanol extract; purple: ethanol.

Initially, one-dimensional ^1^H NMR spectra were recorded to examine the overall metabolomic profiles of each *L. shawii* extract. A stacked plot of one representative NMR spectrum from identical triplicate measurements of each extract is shown in Figure 2, in which a clear distinction can be seen between the extracts depending on the polarity of the solvents used for the extractions, most notably in the sugar region (δ 3.15 to 4.5). The individual spectrum for the LSW extract is provided in Supplementary Figure S1.

**Figure 3:**
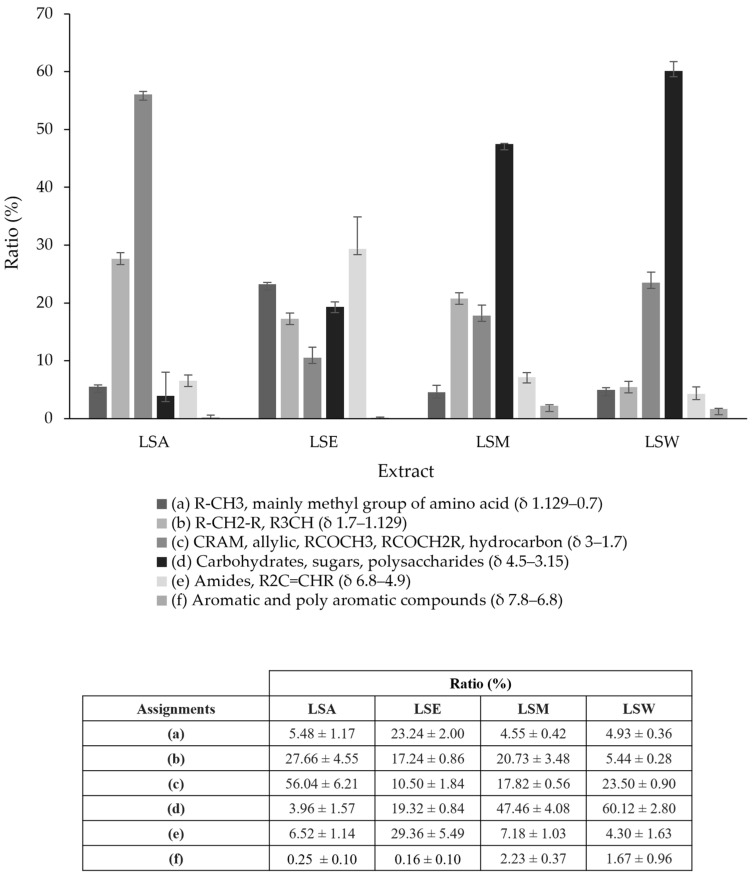
Ratios (%) of six chemical shift assignments calculated from 1D ^1^H NMR spectral integrations for each *L. shawii* extract. The column diagram illustrates that different solvents extract distinct types of metabolites, with details presented in the ratio table. Values are expressed as mean ± SD of triplicate measurements (*n* = 3). Error bars represent the standard deviation of mean values. LSA: acetone extract; LSE: ethanol extract; LSM: methanol extract; LSW: water extract.

The NMR spectra in Figure 2 demonstrate that each solvent extracts distinct types of metabolites. To investigate the approximate composition of the extracts, six chemical shift assignments associated with specific signals were selected from the one-dimensional ^1^H NMR spectra and integrated at (δ 0.7 to 1.129), (δ 1.129 to 1.7), (δ 1.7 to 3), (δ 3.15 to 4.5), (δ 4.9 to 6.8), (δ 6.8 to 7.8), and (δ 7.8 to 9.8). Their respective ratios were then calculated based on the total sum of the integral values and are expressed as percentages. Figure 3 shows a summary of the results.

Overall, sugar-associated signals dominate the spectra, with a significant percentage of signals recorded from the LSW (60.12 ± 2.80), followed by LSM (47.46 ± 4.08), and LSE (19.32 ± 0.84) extracts, while the lowest percentage was recorded from the LSA extract (3.96 ± 1.57). Signals arising from the δ 1.7 to 3 chemical shift region (e.g., RCOCH_2_R, RCH_2_NH_2_, and R_2_C=CRCHR_2_) were recorded with a higher ratio in the LSA extract (56.04 ± 6.21), followed by the LSW extract (23.50 ± 0.90), while a lower ratio was found in the LSM extract (17.82 ± 0.56) and the LSE extract (10.50 ± 1.84). Aromatic-associated signals were detected across all the extracts: the LSM extract showed the highest percentage (2.23 ± 0.37), followed by the LSW extract (1.67 ± 0.96). Negligible amounts of aromatic-associated signals were found in the LSE and LSA extracts. The LSE and LSA extracts expressed the strongest signals in the up-field aliphatic region. It was found that the LSE extract had the most R-CH_3_-associated signals (23.24 ± 2.00), while the LSA extract had the most R-CH_2_-R-associated signals (27.66 ± 4.55). The lowest aliphatic signal percentages were found in the LSM extract (4.55 ± 0.42) and the LSW extract (5.44 ± 0.28). The ethanolic extraction, LSE, recorded a significantly higher percentage of signals in the amides and alkanes region than the rest of the extracts (29.36 ± 5.49). Our results show that the LSM, LSE, and LSW extracts generally share similar features which contrast with the LSA extract, as it is the least polar extract of the four solvents.

**Figure 4:**
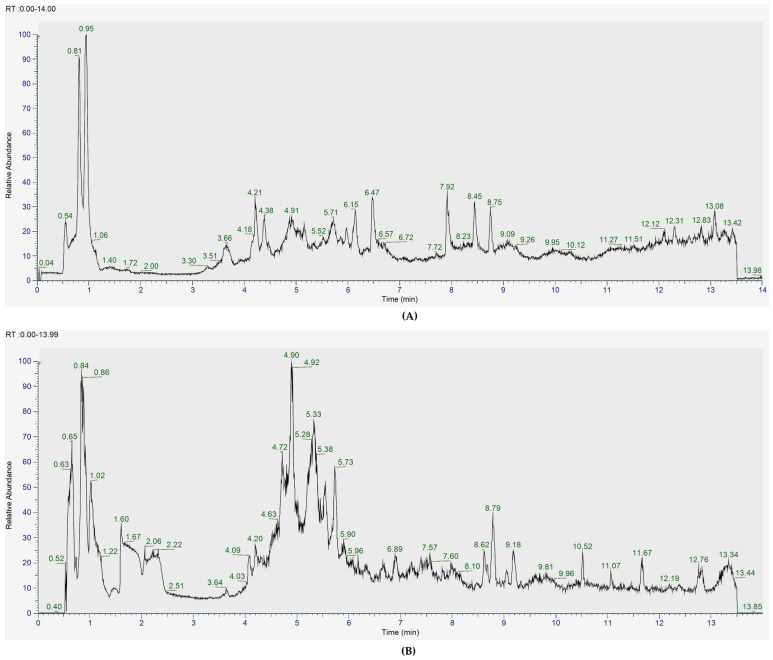
Total ion chromatogram of the pooled samples containing all *L. shawii* extracts obtained using UHPLC–MS in (**A**) ESI+ and (**B**) ESI−modes.

**Figure 5:**
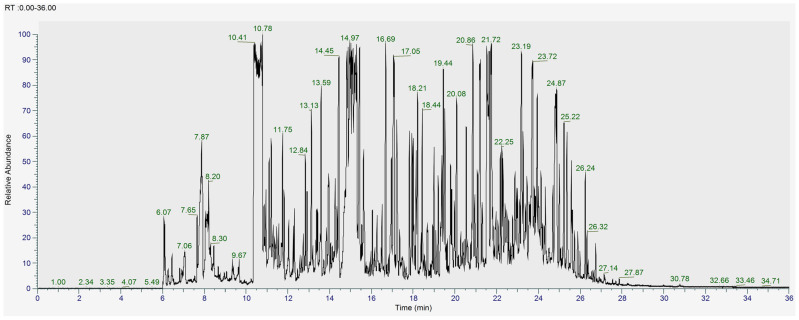
Total ion GC-MS chromatogram of the derivatized pooled sample containing all different solvent *L. shawii* extracts.

To further examine the metabolites of the *L. shawii* extracts, UHPLC-ESI-MS (+/−) and GC-MS were subsequently used for a deeper analysis. Figure 4 and Figure 5 show the total ion chromatograms (TICs) of the pooled *L. shawii* samples obtained using UHPLC-ESI-MS and GC-MS. The individual TICs of the LSW extract per instrument are shown in Supplementary Figures S2–S5.

**Figure 6:**
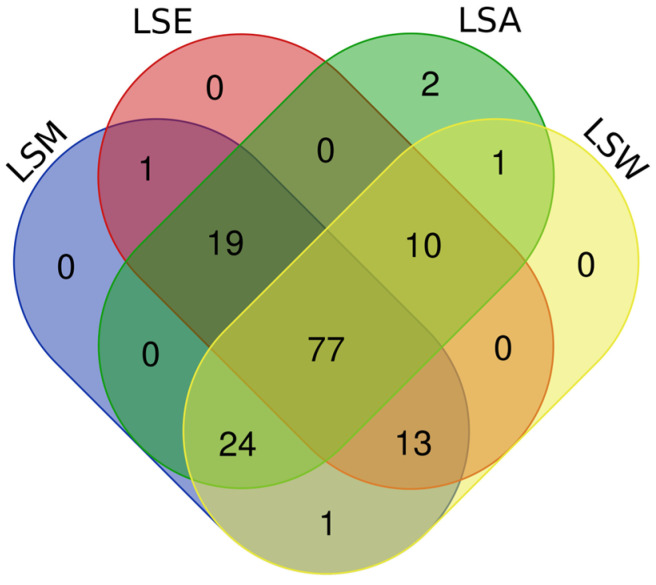
Venn diagram comparing common and unique tentative metabolite identifications between the *L. shawii* extracts using data obtained through GC-MS and UHPLC-ESI-MS (+/−) combined. LSM, methanolic extract; LSE, ethanolic extract; LSA, acetone extract; LSW, water extract.

The results revealed a combined total of 148 unique tentatively annotated features across all the *L. shawii* extracts. The number of features detected in each extract was as follows: 133 in LSA, 120 in LSE, 135 in LSM, and 126 in LSW. Lists of all tentatively identified metabolites and their classifications can be found in Supplementary Tables S1–S3. The Venn diagram shows the distribution of the metabolites detected across the four *L. shawii* extracts (Figure 6). A total of 77 metabolites were common to all the extracts, highlighting a core metabolome that is extractable regardless of solvent. Two unique metabolites were detected in LSA: cholesterol, 1TMS (C_30_H_54_OSi, RT 25.06), and 2-Hydroxybutanedioic acid, 3TMS (C_13_H_30_O_5_Si_3_, RT 10.32). The LSE, LSM, and LSW extracts showed no unique contributions. Further details on the unique and common metabolites across extracts is available in Supplementary Table S4.

Out of the total number of metabolites, 45 unique annotated phytochemicals were tentatively identified in all the *L. shawii* extracts and further analyzed, the majority of which were detected by GC-MS. They included isoprenoids, phenols, phenylpropanoids, flavonoids, alkaloids, benzenoids, quinones, and sterols. Further information on the common and unique phytochemicals in the extracts can be found in Supplementary Table S5.

**Figure 7:**
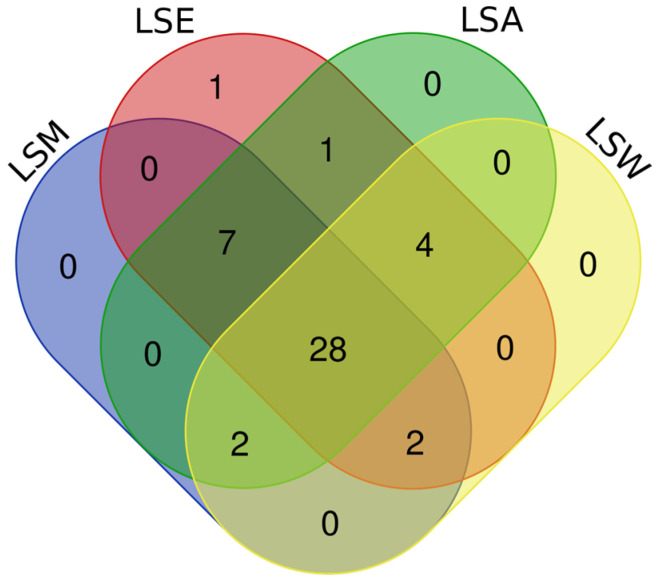
Venn diagram comparing common and unique phytochemicals tentatively detected in the *L. shawii* extracts using data obtained by GC-MS and UHPLC-ESI-MS (+/-) combined. LSM: methanolic extract; LSE: ethanolic extract; LSA: acetone extract; LSW: water extract.

The Venn diagram illustrates the distribution of phytochemicals detected across the four *L. shawii* extracts, revealing a total of 28 phytochemicals common to all the extracts, with LSE having the largest number of phytochemical annotations (43), followed by LSA (42), LSM (39), and lastly LSW (36). Unique compounds were minimal, with only one phytochemical exclusive to LSE. The LSM, LSA, and LSW extracts showed no unique detections. Overlaps among solvents included seven compounds shared by LSM, LSE, and LSA and four shared between LSA and LSW (Figure 7). A summary of the phytochemicals detected is shown in Table 2.

The recorded variations in Table 2 are mainly due to the difference in the extraction solvent polarity, compound volatility, ionization method, and instrument sensitivity and selectivity [37].

**Figure 8:**
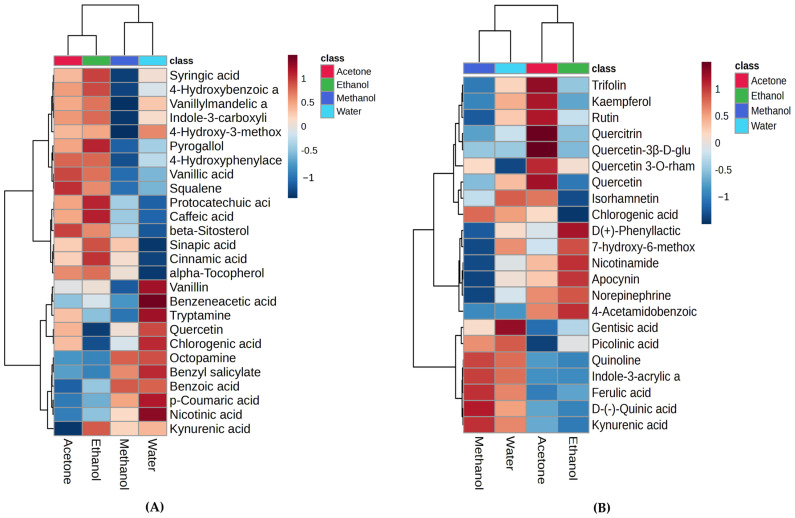
Hierarchical cluster heatmaps of tentatively identified phytochemicals in different *L. shawii* extracts based on ANOVA using (**A**) GC-MS; (**B**) combined UHPLC-ESI-MS (+/−) data. The heatmaps show the average of extract group replicates in the rows and metabolite features in the columns. The color indicates the average concentration of each metabolite, from low (blue) to high (red).

A comparison of the relative abundance of the tentatively identified phytochemicals in each extract is described below using HCA heatmaps. The HCA heatmaps illustrate the variation contained in each sample (Figure 8). The results showed clustering between the aqueous and the methanolic extracts and between the acetone and ethanolic extracts in both MS methods. Further details on the relative abundance of significant phytochemicals and a one-way ANOVA analysis are available in Supplementary Figure S6 and Table S6.

Interestingly, the HCA heatmaps showed that quercetin was detected using both MS methods and had similar trends; it was found at relatively higher levels in the acetone and water *L. shawii* extracts. The aqueous extract had significantly higher levels of gentisic acid, vanillin, benzeneacetic acid, tryptamine, chlorogenic acid, benzyl salicylate, p-coumaric acid, and nicotinic acid than the other extracts. Similar observations can be made for the acetone extract, which had greater levels of trifolin, kaempferol, rutin, quercetrin, quercetin 3β-D-glucoside, and quercetin 3-O-rhamnoside-7-O-glucoside compared to the other extracts. As for the ethanolic extract, it showed relatively higher levels of pyrogallol, squalene, protocatechuic acid, caffeic acid, beta-sitosterol, sinapic acid, cinnamic acid, phenyllactic acid, nicotinamide, and apocynin. Meanwhile, the methanolic extract showed a relative abundance of quinoline, Indole-3-acrylic acid, ferulic acid, quinic acid, and kynurenic acid.

### 3.2. Bioactivities of L. shawii Water Extract

#### 3.2.1. Total Phenolic Content of *L. shawii* Extracts

**Figure 9:**
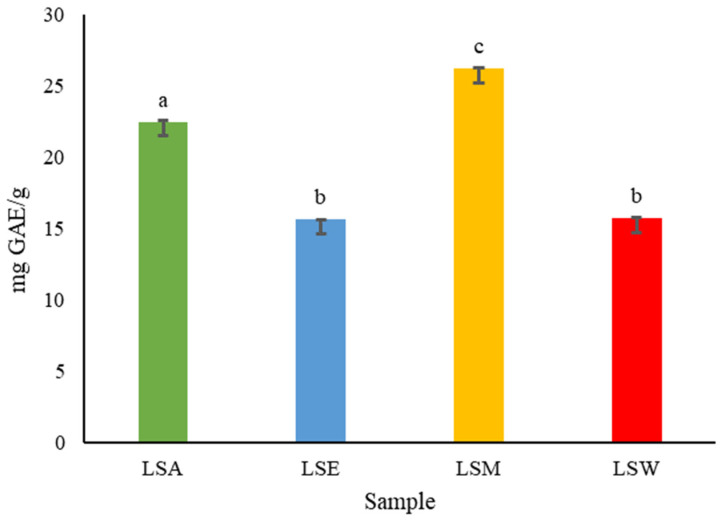
Total phenolic content of *L. shawii* extracts according to solvents used. Values are expressed as mean ± SD (*n =* 3). Different letters (a–c) denote significant differences (*p* < 0.05). LSA: acetone extract; LSE: ethanol extract; LSM: methanol extract; LSW: water extract. GAE: gallic acid equivalent.

Figure 9 shows an estimate of the total phenolic content (TPC) of different solvent extracts of *L. shawii* as determined by the Folin–Ciocalteu method. The highest TPC of *L. shawii* was found in LSM (26.265 ± 0.005 mg GAE/g), followed by LSA (22.50 ± 0.06 mg GAE/g), LSW (15.73 ± 0.02 mg GAE/g), and lastly LSE (15.65 ± 0.01 mg GAE/g). No significant differences (*p* > 0.05) were found between LSE and LSW, indicating that these solvents are similarly efficient at extracting the phenolic compounds present in *L. shawii*.

Solvents of different polarities can solubilize different phenolic compounds based on their own natural polarity and composition, thus influencing the extraction efficiency and resulting TPC. The previous literature supports the conclusion that methanol is considered the most effective due to its high polarity and hydrogen-bonding capacity, allowing it to extract a wider range of phenolic compounds [38]. Previous studies have also reported varying TPC values for *L. shawii*. For instance, a recent study by Kaur et al. (2024) found that the methanolic and aqueous extracts of *L. shawii* collected from India had a TPC of 11.11 mg GAE/g and 6.76 mg GAE/g, respectively [39]. Another study by Ali et al. (2020) reports that *L. shawii* collected from Egypt had TPC values in the range of 55.50 to 230.50 mg GAE/g extract depending on the extraction solvent, in which methanol extracted the highest concentration of phenols [10]. Furthermore, a more recent study shows that the hydroalcoholic extract of *L. shawii* collected from Saudi Arabia contained a TPC of 52.72 mg GAE/g [11]. These variations might be due to the influence of factors such as geographical origin, cultivars, plant maturity, solvent choice, extraction method, and drying method [40,41,42]. It is well known that phenolic compounds exhibit potent antimalarial activity and have the potential to be developed into effective therapeutic agents [43,44]. Therefore, considering that *L. shawii* is commonly consumed as a water decoction in Arabian folk medicine, the LSW extract was subsequently chosen to carry out further investigations of the plant’s bioactivities.

#### 3.2.2. Antibacterial Activity of *L. shawii* Water Extract

Based on a broth microdilution, the LSW extract exhibited weak antibacterial activity against selected clinical isolates, including *P. aeruginosa*, MRSA, *A. baumanii*, and *K. pneumoniae* with MIC values of 500 μg/mL (Table 3).

A study by Hassan et al. (2017) evaluated the antibacterial activity of different solvent extracts of *L. shawii* using the agar diffusion method and found that the chloroform, ethyl acetate, and butanol fractions were active against *S. pneumoniae, B. subtilis*, and *E. coli* but were ineffective against *P. aeruginosa* [17]. A recently published study indicates that the methanolic extract of *L. shawii* may be effective against some Gram-positive and Gram-negative bacteria, namely *E. coli* and *B. cereus,* with inhibition zones of 20.41 and 16.57 mm, respectively, but this study also found that the *L. shawii* extract had little effect on *K. pneumoniae* [45]. Yet another study reports on the antibacterial activity of *L. shawii* seed extracts against a number of bacterial strains, including MRSA isolates, reporting an MIC value of 10 mg/mL [46]. An earlier study reported that different solvent extracts of *L. shawii*, namely aqueous, methanol, and ethyl acetate extracts, showed varying activity against clinical isolates [10]. Furthermore, the ethanolic extract of *L. shawii* has been shown to offer good antibacterial activity using a disc diffusion assay against several bacterial strains [13]. It has also been reported that *L. shawii* extracts have been used as a bio-reductor in synthesizing silver nanoparticles (AgNP) with nanoparticles possessing strong antibacterial activity against selected pathogens [39]. While alcohol extracts offer a more extensive range of bioactive chemicals with different polarities, our findings imply that water extracts might be low in certain less polar compounds (Figure S2). The hydroxyl groups and degree of hydroxylation of phenolic and flavonoid compounds, especially those with antioxidant activity, seem to be closely associated with antimicrobial properties.

#### 3.2.3. In Vitro Antimalarial Activity of *L. shawii* Water Extracts

Potential targets for novel antimalarial medications include the intraerythrocytic stage of the parasite life cycle. During this phase, plasmodium parasites are present inside the human host’s erythrocytes where they start to consume and break down hemoglobin for growth, leading to the release and build-up of free heme and ferriprotoporphyrin (IX) byproducts. These byproducts are poisonous to the parasite and can generate high levels of oxygen radicals [47]; therefore, the plasmodium protects itself by detoxifying the heme by producing hemozoin (β-hematin), a crystalline polymer of ferriprotoporphyrin (IX) [48].

**Figure 10:**
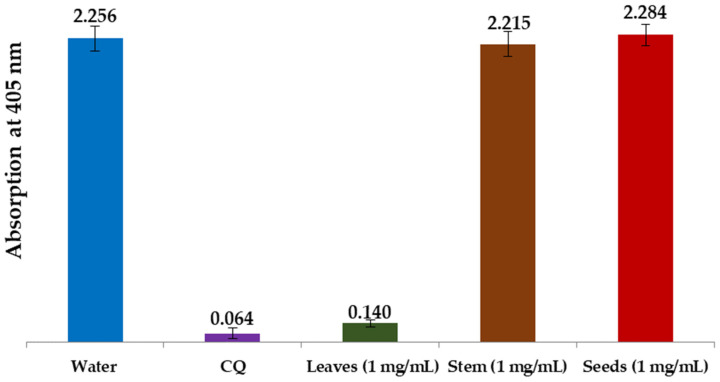
Column diagram representing antimalarial efficiency (absorption of dissolved β-hematin at 405 nm) of water extracts of leaves, stems, and seeds of *L. shawii* compared to negative (water) and positive (CQ-0.1 mg/mL) controls. Each result represents an average of 16 individual experiments.

**Figure 11:**
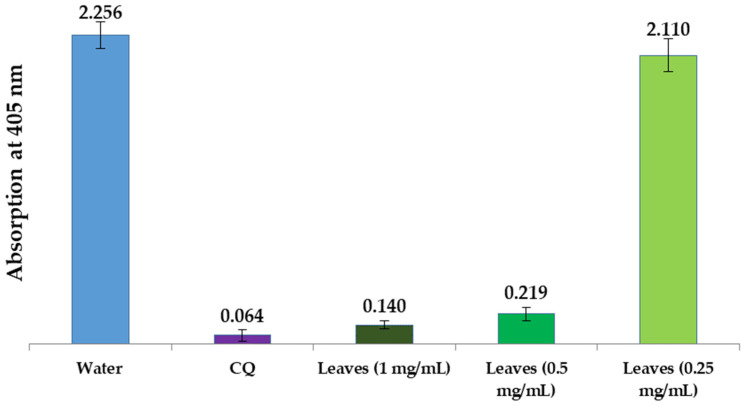
Column diagram representing antimalarial efficiency (absorption of dissolved β-hematin at 405 nm) of different dilutions of the LSW leaf extract, in comparison to negative (water) and positive (CQ-0.1 mg/mL) controls. Each result represents an average of 16 individual experiments.

In the present study, we examined water extracts of the leaves, stems, and seeds of *L. shawii* to identify possible antimalarial properties using a semi-quantitative in vitro assay method. Using positive (CQ-chloroquine 0.1 mg/mL) and negative (water) controls, we compared the effectiveness of these extracts in suppressing β-hematin production in vitro. The results of these semi-quantitative in vitro tests of different parts of the LSW extracts are shown in Figure 10 and Figure 11. As can be seen, the absorption rate is inversely proportional to the efficiency of the extract, i.e., the lower the absorption, the better the extract is in preventing β-hematin formation.

Figure 10 shows that the LSW leaf extract greatly inhibited the formation of β-hematin (with an absorbance of 0.140 ± 0.027), while the stem and seed water extracts were found to be completely inactive as β-hematin inhibitors at an absorbance of 2.215 ± 0.092, and an absorbance of 2.284 ± 0.081, respectively. Figure 11 summarizes the inhibitory effects of different dilutions of LSW leaf extracts (1 mg/mL, 0.5 mg/mL, and 0.25 mg/mL) on β-hematin formation. The inhibition at the highest concentration (with an absorbance of 0.140 ± 0.027) is comparable to the positive control used (with an absorbance of 0.064 ± 0.037), while the level of activity decreases gradually with decreasing concentration until it disappears at the lowest concentration used.

The active flavonoid compounds in these extracts likely produce a complex with ferriheme which inhibits the synthesis of β-hematin, which represents a possible mechanism of inhibition. The results showed that several tentatively identified phytochemicals with reported antimalarial activity were detected in the *L. shawii* leaf extracts (Table 4). According to the HCA analyses, we noted that the LSW and LSM extracts showed relatively higher levels of quinolone, gentisic acid, ferulic acid, vanillin, quercetin, chlorogenic acid, isorhamnetin, and 7-hydroxy-6-methoxy-2H-chromen-2-one, while LSE and LSA exhibited a relatively high abundance of syringic acid, pyrogallol, squalene, protocatechuic acid, caffeic acid, and beta sitosterol. Higher levels of trifolin, kaempferol, rutin, quercitrin, and quercetin were found in LSA. Most importantly, our results might be explained by the particularly higher level of quinolone in the water extract; quinoline and its derivatives, such as chloroquine, have long been recognized and developed as potent antimalarial agents due to their ability to interfere with the heme detoxification pathway in *Plasmodium* parasites [49,50]. Furthermore, one study investigated 42 methanolic extracts of plants from Saudi Arabia, including *L. shawii*, which were assessed for antiplasmodial and anti-trypanosomal activity; three samples exhibited notable antiplasmodial effects, namely the methanolic extract of *L. shawii* at an IC_50_ of 7.750 μg/mL [16].

#### 3.2.4. Anti-Inflammatory Activity of *L. shawii* Water Extracts

**Figure 12:**
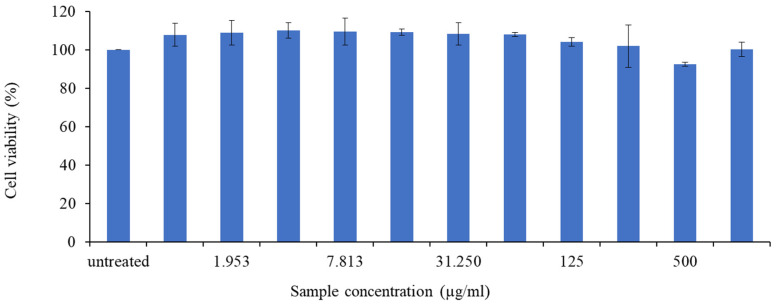
The effects of *L. shawii* water extract on the viability of RAW 264.7 cells. The RAW 264.7 cells were cultured for 24 h in the absence or presence of *L. shawii* water extract at concentrations ranging from 0 to 1000 µg/mL, and then cell viability was evaluated using the MTT method. The data are reported as the means and standard deviations of three replicates.

A preliminary study was carried out to establish the non-toxic concentration of the LSW extract on RAW 264.7 macrophage cells. The RAW 264.7 cells were exposed to the LSW extract at varying concentrations (0–1000 µg/mL) and incubated for 24 h, after which cell viability was assessed using the MTT method, after which we observed that for concentrations up to 1000 µg/mL, the LSW extract did not reduce the viability of the RAW 264.7 cells significantly (Figure 12). These data were then used to determine the sample concentration in the NO inhibition assay on LPS-induced RAW 264.7 cells. The production of NO in macrophages serves as an indicator of inflammation in response to an antigen as nitric oxide is generated by infiltrated cells, damaged tissue, or bacteria, which serve as versatile effectors, enhancing acute inflammation and triggering adaptive immune responses [34]. Therefore, downregulating NO production is an important way to protect against inflammation, which is linked to many diseases. This study was conducted to evaluate the potential of LSW water extract as an anti-inflammatory agent using an NO inhibition assay on LPS-induced RAW 264.8 macrophage-like cells.

**Figure 13:**
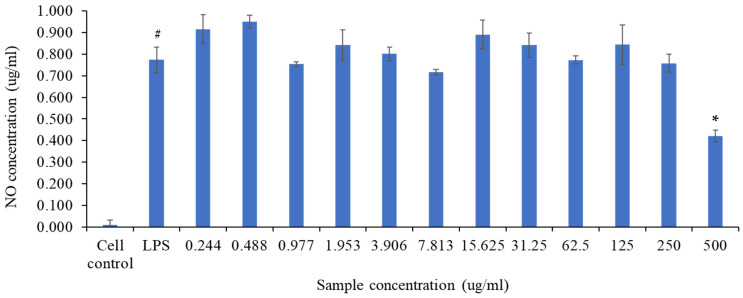
RAW 264.7 cells were treated with *L. shawii* water extract in various concentrations (0.244–500 µg/mL) followed by cell incubation with 1 µg/mL LPS as an inflammatory inducer for 24 h. The supernatant was collected after 24 h, and NO concentration was quantified by Griess assay. Results show that *L. shawii* water extract inhibited NO generation in RAW 264.7 cells at a concentration of 500 µg/mL. The data are represented as the means ± SD (n = 3). ^#^ *p* < 0.05 compared to cell control; * *p* < 0.05 compared to LPS.

The RAW 264.7 cells were incubated with or without LPS (1 μg/mL) as an inflammatory inducer, in the absence or presence of LSW at various concentrations (0.244–500 µg/mL) for 24 h. Lipopolysaccharide (LPS), the primary constituent of the outer membrane of the cell wall of Gram-negative bacteria, can activate macrophages to generate pro-inflammatory cytokines like TNF-α and IL-6, as well as inflammatory mediators such as NO and PGE2. Griess reaction was used to quantify the NO concentrations in the medium culture. The results reveal that incubating cells with the LSW extract for 24 h may decrease NO production in LPS-induced RAW 265.7 cells at a concentration of 500 µg/mL relative to the LPS extract group, as shown in Figure 13.

The results showed that LSW, at the highest concentration (500 µg/mL), decreased NO production, demonstrating moderate-to-weak anti-inflammatory properties. The levels of bioactive compounds in the aqueous extract that contribute to the anti-inflammatory activity might be low according to our metabolomics analysis, while the other organic extracts contained a more diverse phytochemical profile. Table 5 shows the presence of phytochemicals with known anti-inflammatory properties in the *L. shawii* extracts.

According to the HCA analyses, the LSW extract was relatively rich in vanillin, quercetin, chlorogenic acid, and gentisic acid. The LSW and LSM extracts both exhibited a relatively higher abundance of octopamine, p-coumaric acid, quinoline, ferulic acid, and kynurenic acid, while LSA and LSE showed relatively higher levels of syringic acid, pyrogallol, squalene, protocatechuic acid, caffeic acid, beta-Sitosterol, sinapic acid, and cinnamic acid.

The nuclear factor-kappa B (NF-κB) transcription factor is important in immune and inflammatory responses by controlling the expression of genes for pro-inflammatory cytokines, adhesion molecules, chemokines, growth factors, and enzymes like cyclooxygenase-2 (COX-2) and inducible nitric oxide synthase (iNOS). In this context, several studies have reported on the anti-inflammatory activities of *L. shawii* extracts, such as, for example, that of Alkuwari et al. (2010), who reported that the fractionated methanol extract of *L. shawii* may inhibit NF-kβ activation in NF-κB-luciferase reporter HEK293 [113]. Another study also revealed that *L. shawii* ethyl acetate extract is more efficient at inhibiting COX-2 activity compared to the anti-inflammatory drug aspirin in the sera of thyroid cancer patients [15].

It is worth noting that the initial immune response, i.e., the production of cytokines, during malarial infection is vital to controlling parasite growth. If this inflammation becomes excessive and dysregulated, it can significantly contribute to the development of severe malaria, such as cerebral malaria, potentially leading to death [114], and comprehensive treatment therefore involves addressing both the disease itself, i.e., the parasite reproduction cycles, and its symptoms, such as inflammation. 

## 4. Conclusions

In the current study, we utilized NMR spectroscopy, GC-MS, and UHPLC-MS to achieve a comprehensive profile of metabolites from *L. shawii* leaves extracted using five different solvents, and then we evaluated the bioactivities of the LSW extract in vitro. A total of 148 unique features were tentatively identified across the *L. shawii* extracts. The LSE extract contained the highest number of unique metabolites (22), while LSA had the fewest (5). A further analysis tentatively identified 45 phytochemicals across the extracts, with LSE having the largest number of phytochemical annotations (43), followed by LSA (42), LSM (39), and lastly LSW (36). The extracts contained diverse classes of phytochemicals, with phenylpropanoids, flavonoids, alkaloids, and benzenoids being the most prevalent. Additionally, the solvent polarity influenced the extraction efficiency, impacting the total phenolic content (TPC) of the extracts as evidenced by the profiling results. The highest total phenolic content was found in LSM (26.265 ± 0.005 mg GAE/g). Based on the traditional use of *L. shawii* infusions in Arabian folk medicine, a more detailed evaluation of the bioactivity of LSW was carried out. In this part of our study, we found that LSW showed weak antibacterial activity against *P. aeruginosa*, MRSA, *A. baumannii*, and *K. pneumoniae*, with MIC values of 500 µg/mL. Furthermore, LSW demonstrated generally weak anti-inflammatory effects, evidenced by the reduction in NO production in LPS-induced RAW 264.7 macrophage cells at 500 µg/mL. Focusing on antimalarial activity, the *L. shawii* leaf extract (1 mg/mL) demonstrated potent β-hematin inhibition (at an absorbance of 0.140 ± 0.027) comparable to the chloroquine positive control (at an absorbance of 0.064 ± 0.037), suggesting water-based *L. shawii* extracts may have promising antimalarial potential. This activity may be linked to the relatively abundant presence of quinoline, an alkaloid that has long been utilized as a potent antimalarial agent, in the water extract, which has been known to inhibit β-hematin synthesis by binding to ferriheme. Other phytochemicals with reported antimalarial activity have also been identified with a relatively high abundance in LSW, such as gentisic acid, ferulic acid, vanillin, quercetin, chlorogenic acid, isorhamnetin, and 7-hydroxy-6-methoxy-2H-chromen-2-one. We may therefore conclude that these preliminary results showcase the significant antimalarial activity of the crude water extract of *L. shawii,* which is linked to diverse tentatively identified bioactive phytochemicals. This lays the groundwork for further investigations, including targeted metabolomic studies and bio-assay-guided fractionation, to quantify these compounds and isolate those that have the most potential to be developed as antimalarial agents.

## Figures and Tables

**Table 1:** Botanical profile of *Lycium shawii* and its ethnomedicinal uses.

Botanical Name	*Lycium shawii* Roem & Schult
Local name	Awsaj
Family	Solanaceae
Habitat	Semi-desert, extreme desert, and arid environment
Distribution	Africa, Middle East, Indian subcontinent
Medicinal uses	To treat mouth ulcers, eye ailments, cough, backache, jaundice, constipation, and stomach ache; to act as a hypotensive in humans; and to cure tick fever in livestock
References	[8,9,19]

**Table 2:** Summary of the main classes and subclasses of the tentatively identified phytochemicals in the *L. shawii* extracts according to method used. A: acetone; E: ethanol; M: methanol; W: water.

Tentative Phytochemicals			Method	Extract
Isoprenoids	(a) C30 isoprenoids	Squalene	GC-MS	A, E, M
Phenols	(a) Methoxyphenols	4-Hydroxy-3-methoxyphenylglycol	GC-MS	A, E, M, W
	(b) Benzenetriols	Pyrogallol	GC-MS	A, E, W
Phenylpropanoids	(a) Cinnamic acids	4-Hydroxyphenylacetic acid	GC-MS	A, E, M, W
		Caffeic acid	GC-MS	A, E, M, W
		Benzeneacetic acid	GC-MS	A, E, M, W
		Chlorogenic acid	GC-MS, UHPLC-ESI-MS (−)	A, E, M, W
		Ferulic acid	GC-MS, UHPLC-ESI-MS (+)	A, E, M, W
		Cinnamic acid	GC-MS	A, E, M
		Sinapic acid	GC-MS	A, E, M
		p-Coumaric acid	GC-MS	E, M, W
		Phenyllactic acid	UHPLC-ESI-MS (−)	A, E, M, W
		Vanillylmandelic acid	GC-MS	A, E, W
Flavonoids	(a) Flavonols	Quercetin	GC-MS, UHPLC-ESI-MS (+)	A, E, M, W
		Quercetin 3-O-rhamnoside-7-O-glucoside	UHPLC-ESI-MS (+)	A, E, M, W
		Quercetin-3β-D-glucoside	UHPLC-ESI-MS (+)	A, E, M, W
		Quercitrin	UHPLC-ESI-MS (+)	A, E, M, W
		Kaempferol	UHPLC-ESI-MS (+)	A, E, M, W
		Isorhamnetin	UHPLC-ESI-MS (+)	A, E, M, W
		Rutin	UHPLC-ESI-MS (−)	A, E, M, W
		Trifolin	UHPLC-ESI-MS (+)	A, E, M, W
	(b) Coumarins	7-hydroxy-6-methoxy-2H-chromen-2-one	UHPLC-ESI-MS (+)	A, E
Alkaloids	(a) Tryptophan alkaloids	Tryptamine	GC-MS	A, E, M, W
		Indole-3-acrylic acid	UHPLC-ESI-MS (+)	A, E, M, W
		Indole-3-carboxylic acid	GC-MS	A, E, M
	(b) Anthranilic acid alkaloids	Kynurenic acid	GC-MS, UHPLC-ESI-MS (+)	A, E, M, W
	(c) Tyrosine alkaloids	Octopamine	GC-MS	A, E, M, W
		Norepinephrine	UHPLC-ESI-MS (+)	A, E, M, W
	(d) Pyridine alkaloids	Nicotinic acid	GC-MS	A, E, M, W
		Nicotinamide	UHPLC-ESI-MS (+)	A, E
		Picolinic acid	UHPLC-ESI-MS (+)	A, E, M, W
	(e) Quinazoline alkaloids	Quinoline	UHPLC-ESI-MS (+)	A, M, W
Benezenoids	(a) Hydroxybenzoic acids	4-Hydroxybenzoic acid	GC-MS	A, E, M, W
		Benzoic acid	GC-MS	A, E, M, W
		Vanillic acid	GC-MS	A, E, M, W
		Benzyl salicylate	GC-MS	E, M, W
		Protocatechuic acid	GC-MS	A, E, M
		Gentisic acid	UHPLC-ESI-MS (−)	A, E, M, W
		Vanillin	GC-MS	A, E, W
		Apocynin	UHPLC-ESI-MS (+)	A, E, M, W
		Quinic acid	UHPLC-ESI-MS (−)	A, M, W
		Syringic acid	GC-MS	A, E, W
	(b) Acylaminobenzoic acids	4-Acetamidobenzoic acid	UHPLC-ESI-MS (−)	A, E, M
Quinones	(a) Quinones	alpha-Tocopherol	GC-MS	A, E, M
Sterols	(a) Stigmasterols	beta-Sitosterol	GC-MS	E

**Table 3:** Antibacterial MIC results of LSW extract.

Sample	MIC (μg/mL)			
	*P. aeruginosa*	MRSA	*A. baumannii*	*K. pneumoniae*
LSW extract	500	500	500	500
Vancomycin	62.5	1.95	125	62.5

**Table 4:** Summary of tentatively identified phytochemicals in the *L. shawii* extracts and their reported anti-malarial activities. Data obtained from GC-MS data are TMS-derivatized. A: acetone; E: ethanol; M: methanol; W: water; RT, retention time; RI, retention index; *m/z*, mass-to-charge ratio.

			GC-MS			UHPLC-ESI-MS				
No.	Phytochemical Name	Extract	RT (min)	Calc. RI	Delta RI	RT (min)	Annot. DeltaMass (ppm)	*m/z*	Adduct	Antimalarial Activity
1	beta-Sitosterol	E	26.24	3344	20	–	–	–	–	[51]
2	Syringic acid	A, E, W	15.22	1889	6	–	–	–	–	[52,53]
3	Squalene	A, E, M	22.845	2811	0	–	–	–	–	[54]
4	Pyrogallol	A, E, W	12.035	1596	78	–	–	–	–	[55,56]
5	Vanillin	A, E, W	12.607	1647	14					[57,58]
6	Protocatechuic acid	A, E, M	14.422	1813	16	–	–	–	–	[59]
7	Caffeic acid	A, E, M, W	17.5	2133	17	–	–	–	–	[60]
8	Ferulic acid	A, E, M, W	17.139	2092	160	–	–	–	–	[60]
9	Chlorogenic acid	A, E, M, W	25.112	3106	3	4.625	−0.93	353.08751	[M−H] ^−1^	[60,61]
10	Quercetin	A, E, M, W	25.252	3177	43	5.509	0.32	303.05004	[M+H]^+1^	[62,63,64,65]
11	Quercitrin	A, E, M, W	–	–	–	5.745	0.34	449.10799	[M+H]^+1^	[65]
12	Rutin	A, E, M, W	–	–	–	5.307	1.54	609.14705	[M−H]^−1^	[64,66,67]
13	Kaempferol	A, E, M, W	–	–	–	5.988	0.455	287.05514	[M+H]^+1^	[68,69]
14	Trifolin	A, E, M, W	–	–	–	5.978	0.45	449.10804	[M+H]^+1^	[70,71]
15	Isorhamentin	A, E, M, W	–	–	–	6.018	0.43	317.06572	[M+H]^+1^	[72]
16	7-hydroxy-6-methoxy-2H-chromen-2-one isomer 1	A, E	–	–	–	4.813	−0.04	193.04953	[M+H]^+1^	[73]
17	7-hydroxy-6-methoxy-2H-chromen-2-one isomer 2	A, E	–	–	–	5.935	−0.13	193.04951	[M+H]^+1^	[73]
18	Quinoline	A, M, W	–	–	–	6.168	0.46	130.06518	[M+H]^+1^	[49]
19	Gentisic acid	A, E, M, W	–	–	–	8.671	0.56	153.01942	[M−H] ^−1^	[74]

**Table 5:** Summary of tentatively identified phytochemicals in the *L. shawii* extracts and their reported anti-inflammatory activities. GC-MS data are TMS-derivatized. A: acetone; E: ethanol; M: methanol; W: water; RT, retention time; RI, retention index; *m/z*, mass-to-charge ratio.

			GC-MS			UHPLC-ESI-MS				
No.	Phytochemical Name	Extract	RT (min)	Calc. RI	Delta RI	RT (min)	Annot. DeltaMass (ppm)	*m/z*	Adduct	Anti-Inflammatory Activity
1	beta-Sitosterol	E	26.24	3344	20	–	–	–	–	[75]
2	Syringic acid	A, E, W	15.22	1889	6	–	–	–	–	[76]
3	Squalene	A, E, M	22.845	2811	0	–	–	–	–	[77]
4	Pyrogallol	A, E, W	12.035	1596	78	–	–	–	–	[78,79]
5	Vanillin	A, E, W	12.607	1647	14	–	–	–	–	[80,81]
6	Protocatechuic acid	A, E, M	14.422	1813	16	–	–	–	–	[82]
7	Caffeic acid	A, E, M, W	17.5	2133	17	–	–	–	–	[83,84]
8	Ferulic acid	A, E, M, W	17.139	2092	160	–	–	–	–	[85,86]
9	Chlorogenic acid	A, E, M, W	25.112	3106	3	4.625	−0.93	353.08751	[M−H] −^1^	[87,88]
10	Quercetin	A, E, M, W	25.252	3177	43	5.509	0.32	303.05004	[M+H]^+1^	[89,90,91]
11	Quercitrin	A, E, M, W	–	–	–	5.745	0.34	449.10799	[M+H]^+1^	[92]
12	Rutin	A, E, M, W	–	–	–	5.307	1.54	609.14705	[M−H] ^−1^	[92,93,94,95]
13	Kaempferol	A, E, M, W	–	–	–	5.988	0.455	287.05514	[M+H]^+1^	[96,97]
15	Isorhamentin	A, E, M, W	–	–	–	6.018	0.43	317.06572	[M+H]^+1^	[97,98]
16	Apocynin	A, E, M, W				0.99	−0.04	167.07026	[M+H]^+1^	[99,100]
17	Gentisic acid	A, M, W	–	–	–	6.168	0.46	130.06518	[M+H]^+1^	[101]
18	p-Coumaric acid	E, M, W	15.6935	1933	13	–	–	–	–	[102]
19	Sinapic acid	A, E, M	18.443	2242	33	–	–	–	–	[103,104]
20	Cinnamic acid	A, E, M	11.49	1550	3	–	–	–	–	[105]
21	Octopamine	A, E, M, W	15.55	1923	186	–	–	–	–	[106]
22	Kynurenic acid	A, E, M, W	16.872	2064	22	6.102	−0.06	190.04986	[M+H]^+1^	[107]
23	7-hydroxy-6-methoxy-2H-chromen-2-one isomer 1	A, E	–	–	–	4.813	−0.04	193.04953	[M+H]^+1^	[108,109]
24	7-hydroxy-6-methoxy-2H-chromen-2-one isomer 2	A, E	–	–	–	5.935	−0.13	193.04951	[M+H]^+1^	[108,109]
25	Quinoline	A, M, W	–	–	–	6.168	0.46	130.06518	[M+H]^+1^	[110]
25	Quercetin 3-O-rhamnoside-7-O-glucoside	A, E, M, W	–	–	–	6.191	0.37	611.16089	[M+H]^+1^	[111]
25	Quercetin-3β-D-glucoside isomer 1	A, E, M, W	–	–	–	5.75	0.11	465.1028	[M+H]^+1^	[112]
25	Quercetin-3β-D-glucoside isomer 2	A, E, M, W	–	–	–	6.189	0.38	465.10293	[M+H]^+1^	[112]

## Data Availability

The GC-MS and UHPLC-MS original data presented in the study were deposited in the MetaboLights database (https://www.ebi.ac.uk/metabolights/, accessed on 22 October 2024) under the study identifier MTBLS10952.

## Supplementary Materials

The following supporting information can be downloaded at: https://www.mdpi.com/article/10.3390/metabo15020084/s1, Figure S1. ^1^H NMR spectrum of *L. shawii* water extract from δ 0 to 10. Extended view of chemical shift regions of (B) δ 0.5 to 2.7; (C) δ 2.5 to 5.5; (D) δ 5.5 to 8.6. Spectra were acquired at 800 MHz. Figure S2. Extended views of 1H NMR spectra of *L. shawii* water (blue) and ethanol (red) extract of (A) aliphatic and (B) aromatic chemical shift regions showcasing differences in composition. Spectra were acquired at 800 MHz. Figure S3. Total ion chromatogram of *L. shawii* water extract obtained using UHPLC–MS in ESI+. Figure S4. Total ion chromatogram of *L. shawii* water extract, obtained using UHPLC–MS in ESI- mode. Figure S5. Total ion chromatogram of derivatized *L. shawii* water extract obtained using GC-MS. Figure S6. Post-hoc analysis following ANOVA highlighting the relative abundance of significant phytochemicals abundance among *L. shawii* extracts according to method used. Table S1. RefMet nomenclature and chemical classifications of total tentatively identified metabolites (148 metabolites) obtained using The Metabolomics Workbench online tool (https://www.metabolomicsworkbench.org/, accessed on 24 November 2024). Table S2. Tentatively identified metabolites using UHPLC-ESI-MS (+/−) data. Table S3. Tentatively identified TMS-derivatized metabolites using GC-MS data. SI, Similarity Index; RI, retention index. Table S4. Venn analysis results displaying the total unique and common tentative metabolites between the extracts, as shown in Figure 6. Table S5. Venn analysis results displaying unique and common tentatively identified phytochemicals between instruments, as shown in Figure 8. Table S6. ANOVA analysis (*p* < 0.05) showing the significant tentative phytochemicals that contribute to the separation among extracts, as shown in Figure S6.
