# Supplementary material for: Comprehensive Metabolomics Profiling and Bioactivity Study of Lycium shawii (Awsaj) Extracts with Particular Emphasis on Potential Anti-Malarial Properties
Source: Metabolites. 2025 Feb 1;15(2):84. doi: 10.3390/metabo15020084 (PMC11857410; doi:10.3390/metabo15020084)
Supplement: Supplementary file 1 [file metabolites-15-00084-s001.zip › metabolites-3288312-supplementary.pdf]

metabolites

MDPI

Supplementary Materials

Comprehensive Metabolomics Profiling and Bioactivity Study of *Lycium shawii* (Awsaj) Extracts with Particular Emphasis on Potential Anti-Malarial Properties

Ruba Al-Nemi [1], Mutaz Akkawi [2], Khalid Sawalha [2], Siska Andrina Kusumastuti [3], Nuralih [3], Susi Kusumaningrum [3], Tia Okselni [3], Vania Chlarisa Situmorang [3], Abdi Wira Septama [3], Mariusz Jaremko [1,*] and Abdul-Hamid Emwas [4,*]

[1] Bioscience Program, Biological and Environmental Sciences and Engineering Division, King Abdullah University of Science and Technology (KAUST), Thuwal 23955-6900, Saudi Arabia; ruba.nami@kaust.edu.sa
[2] Life Sciences Department, Faculty of Science & Technology, Al-Quds University, Jerusalem P.O. Box 20002, Palestine; makkawi@staff.alquds.edu (M.A.); ksawalha@staff.alquds.edu (K.S.)
[3] Research Center for Pharmaceutical Ingredients and Traditional Medicine, National Research and Innovation Agency, Cibinong, Kabupaten Bogor 16911, Indonesia; sisk001@brin.go.id (S.A.K.); nura008@brin.go.id (N.); susi004@brin.go.id (S.K.); tia.okselni@gmail.com (T.O.); vania.s@gmail.com (V.C.S.); abdi001@brin.go.id (A.W.S.)
[4] KAUST Core Laboratories, King Abdullah University of Science and Technology, Thuwal 23955-6900, Saudi Arabia
* Correspondence: mariusz.jaremko@kaust.edu.sa (M.J.); abdelhamid.emwas@kaust.edu.sa (A.-H.E.)

metabolites

MDPI

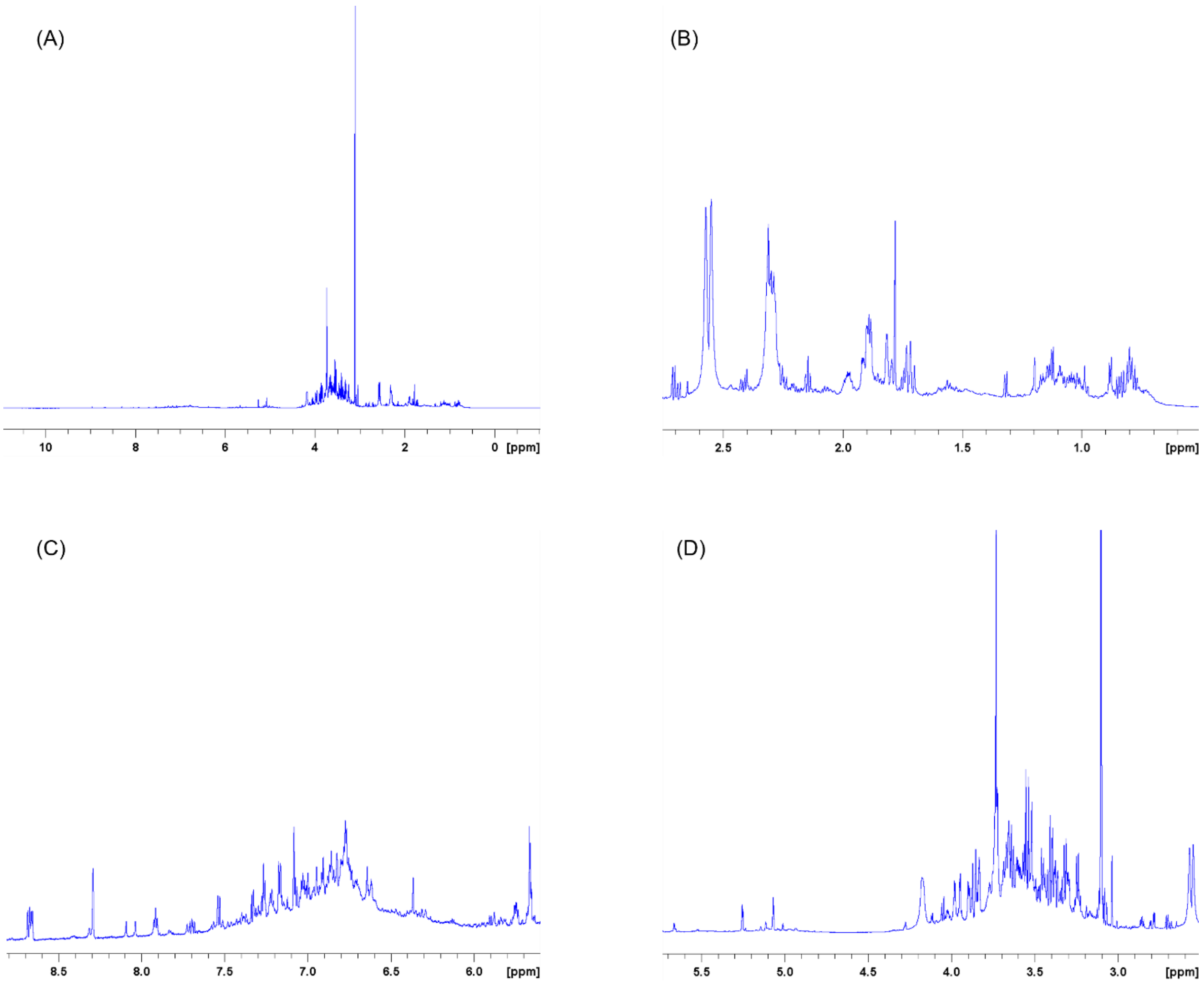

Figure S1. ^{1}H NMR spectrum of *L. shawii* water extract from δ 0 to 10. Extended view of chemical shift regions of (B) δ 0.5 to 2.7; (C) δ 2.5 to 5.5; (D) δ 5.5 to 8.6. Spectra were acquired at 800 MHz.

metabolites

MDPI

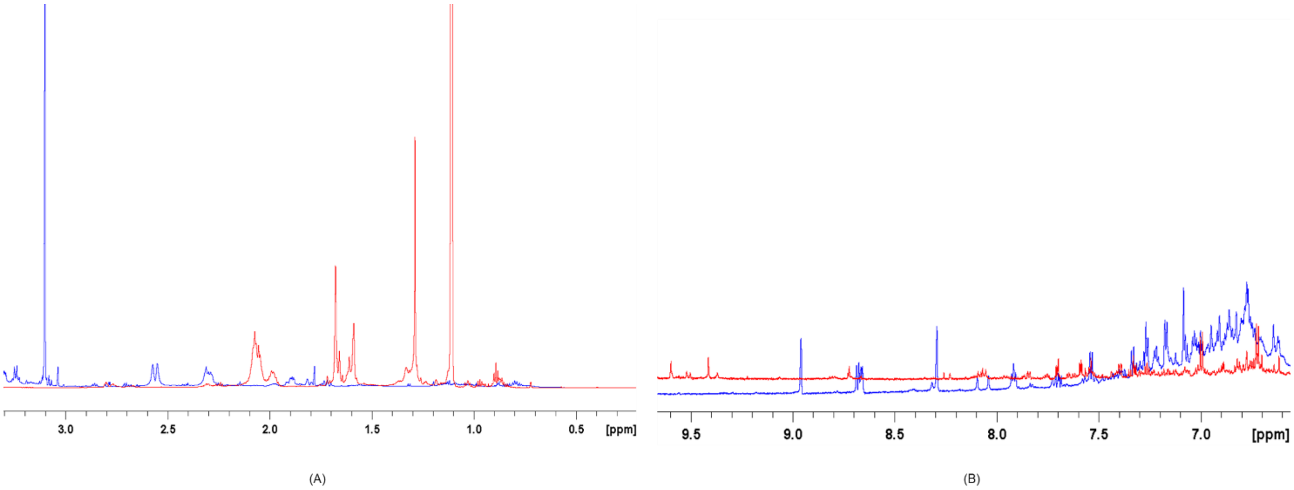

Figure S2. Extended views of ^{1}H NMR spectra of *L. shawii* water (blue) and ethanol (red) extract of (**A**) aliphatic and (**B**) aromatic chemical shift regions showcasing differences in composition. Spectra were acquired at 800 MHz.

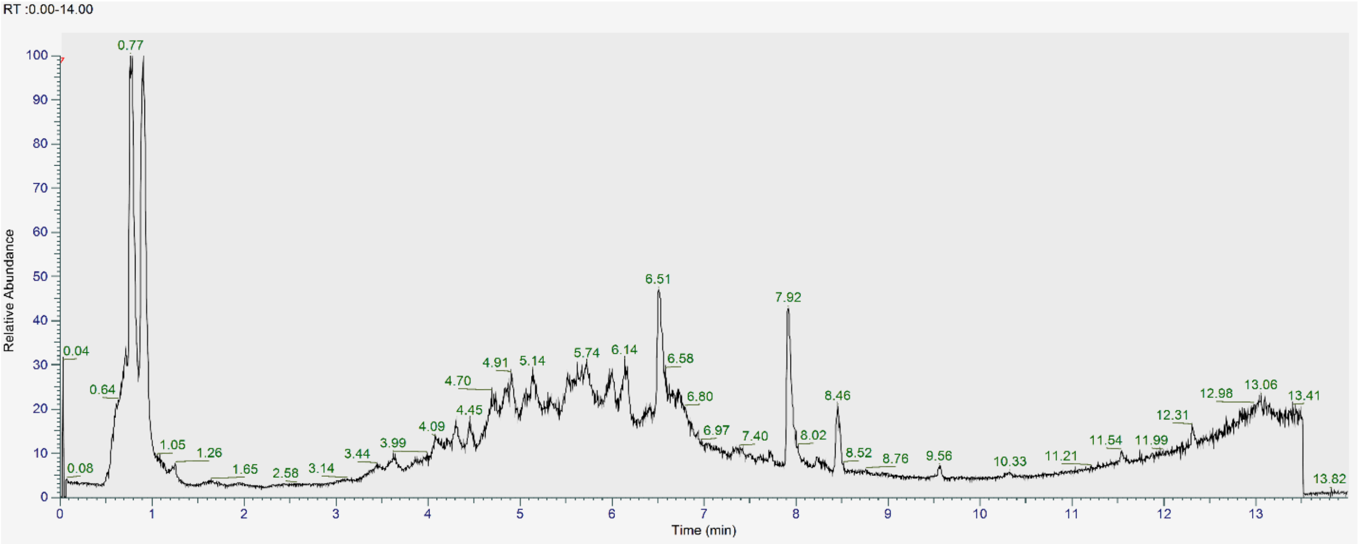

Figure S3. Total ion chromatogram of *L. shawii* water extract obtained using UHPLC–MS in ESI+

mode.

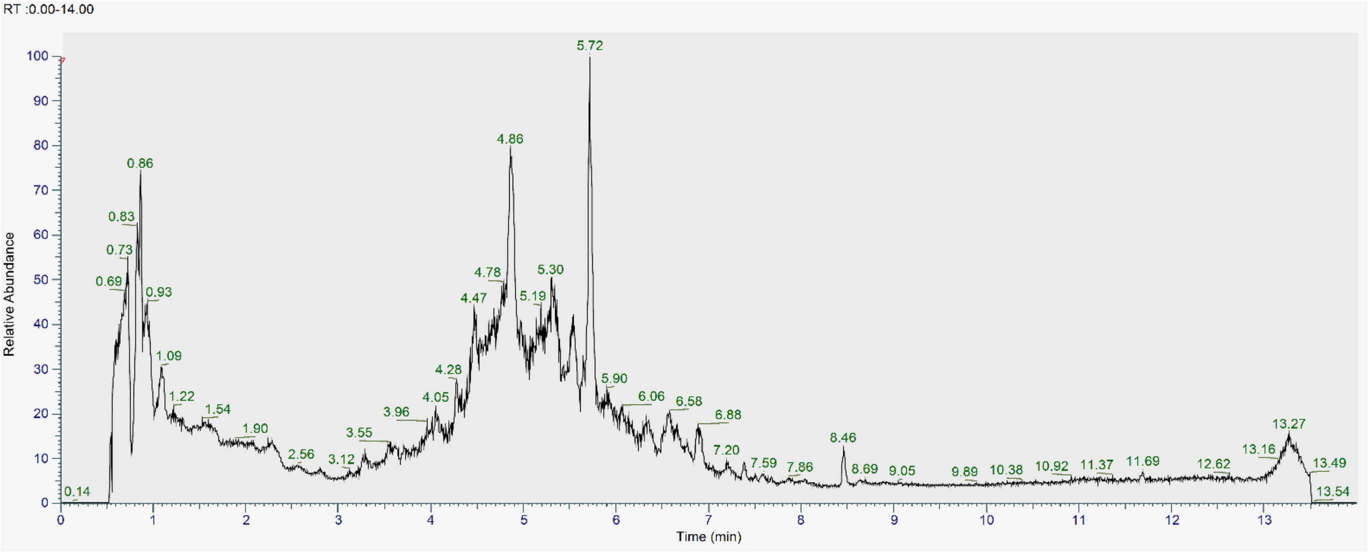

Figure S4. Total ion chromatogram of *L. shawii* water extract, obtained using UHPLC–MS in ESI- mode.

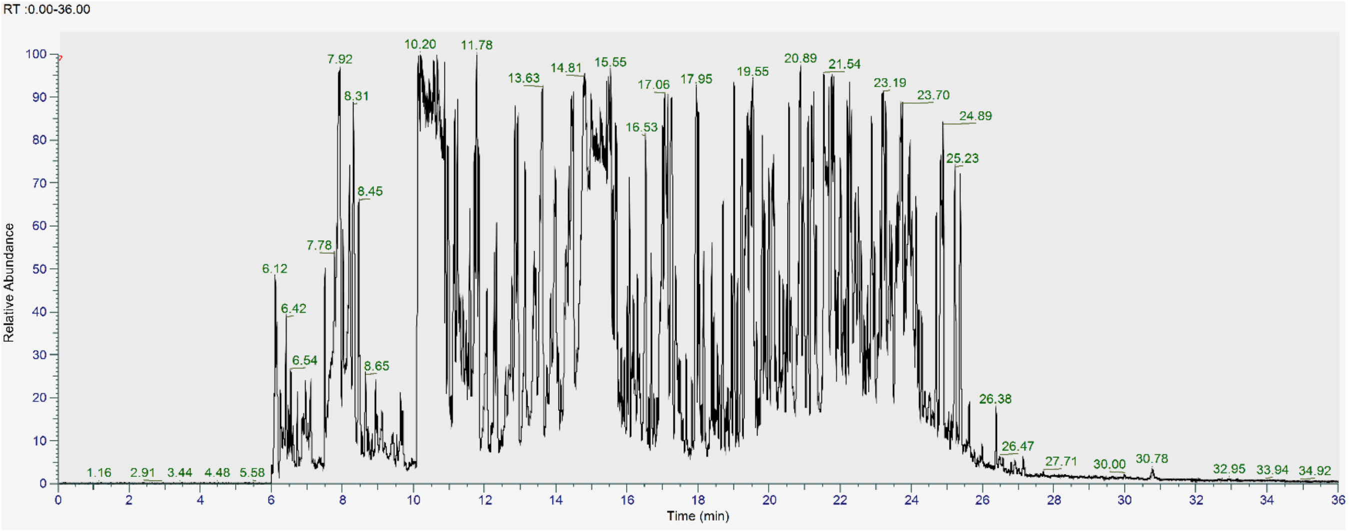

Figure S5. Total ion chromatogram of derivatized *L. shawii* water extract obtained using GC-MS.

metabolites

MDPI

(A) GCMS

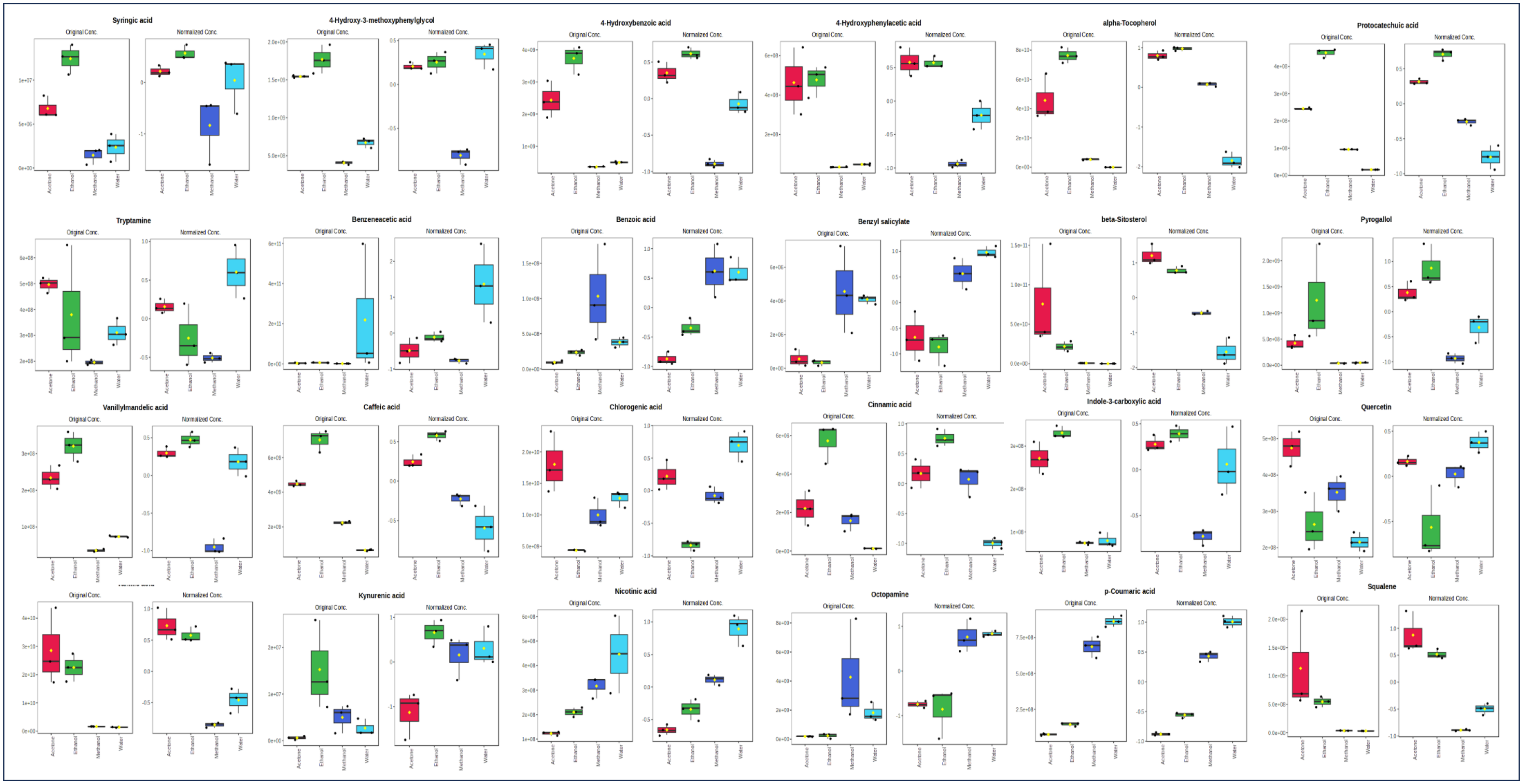

(B) UHPLC-ESI-MS (+/-)

metabolites

MDPI

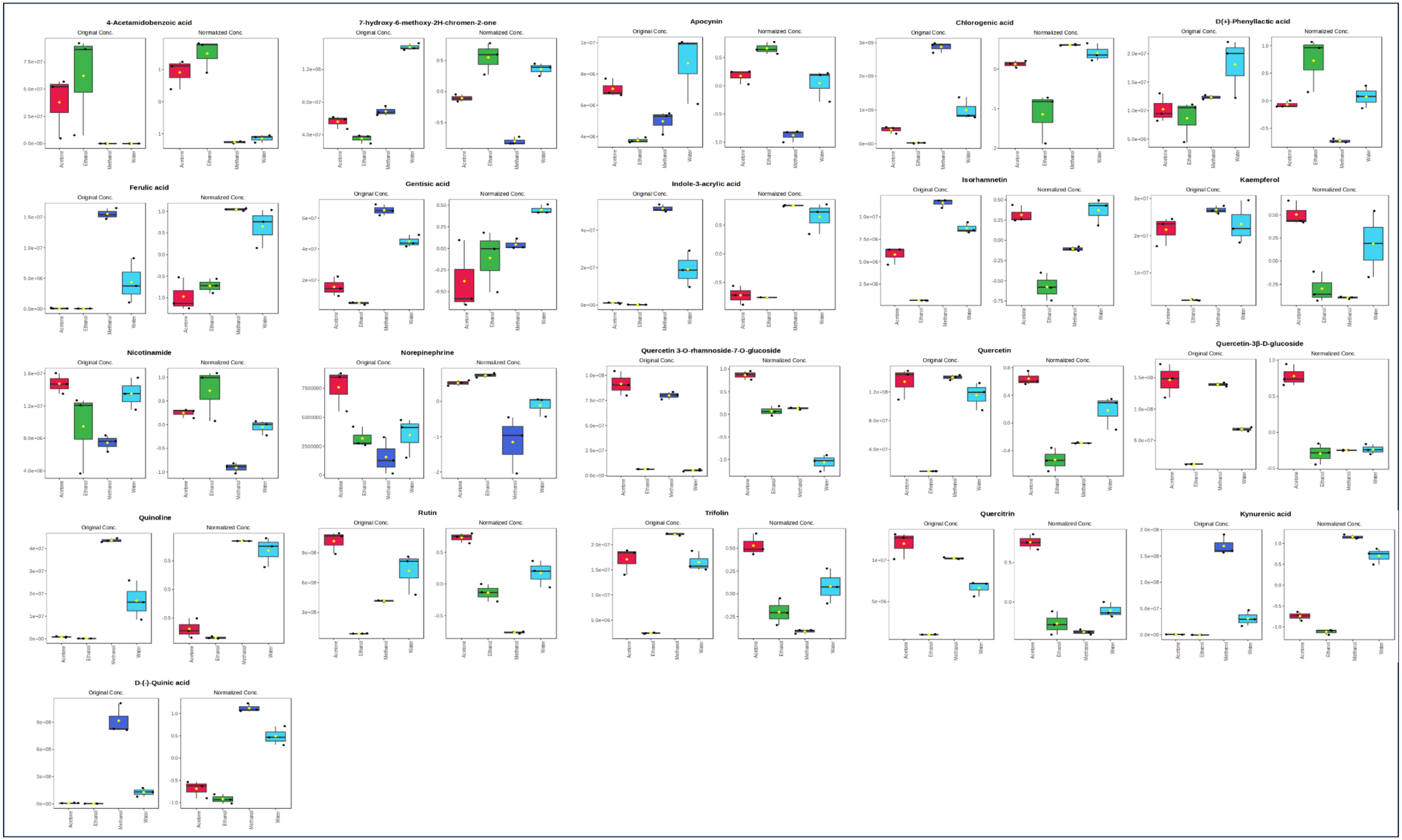

Figure S6. Post-hoc analysis following ANOVA highlighting the relative abundance of significant phytochemicals abundance among *L. shawii* extracts according to method used.

Table S1. RefMet nomenclature and chemical classifications of total tentatively identified metabolites (148 metabolites) obtained using The Metabolomics Workbench online tool (https://www.metabolomicsworkbench.org/).

Input name	Standardized name	Formula	Exact mass	Super class	Main class	Sub class
quercetin-3β-d-glucoside	-					
Xylose	Xylose	150.0528	$C_5H_{10}O_5$	Carbohydrates	Monosaccharides	Pentoses
Kaempferol	Kaempferol	286.0477	$C_{15}H_{10}O_6$	Polyketides	Flavonoids	Flavones and Flavonols
Gluconolactone	Gluconolactone	178.0477	$C_6H_{10}O_6$	Carbohydrates	Monosaccharides	Monosaccharides
Tyrosine	Tyrosine	181.0739	$C_9H_{11}NO_3$	Organic acids	Amino acids and peptides	Amino acids
4-Hydroxy-3-methoxyphenylglycol	Vanylglycol	184.0736	$C_9H_{12}O_4$	Benzenoids	Phenols	Methoxyphenols
Apocynin	Apocynin	166.063	$C_9H_{10}O_3$	Benzenoids	Benzenes	Hydroxybenzoic acids
D(+)-Phenyllactic acid	L-3-Phenyllactic acid	166.063	$C_9H_{10}O_3$	Organic acids	Phenylpropanoids	Cinnamic acids
Isorhamnetin	Isorhamnetin	316.0583	$C_{16}H_{12}O_7$	Polyketides	Flavonoids	Flavones and Flavonols
Caffeic acid	3,4-Dihydroxy-trans-cinnamate	180.0423	$C_9H_8O_4$	Organic acids	Phenylpropanoids	Cinnamic acids
Glyceric acid	Glyceric acid	106.0266	$C_3H_6O_4$	Carbohydrates	Monosaccharides	Sugar acids
Glucose	Glucose	180.0634	$C_6H_{12}O_6$	Carbohydrates	Monosaccharides	Hexoses
Quercetin	Quercetin	302.0427	$C_{15}H_{10}O_7$	Polyketides	Flavonoids	Flavones and Flavonols
Proline	Proline	115.0633	$C_5H_9NO_2$	Organic acids	Amino acids and peptides	Amino acids
1,3,5-trihydroxy-4-{[(2E)-3-(3-hydroxy-4-methoxyphenyl)prop-2-enoyl]oxy}cyclohexane-1-carboxylic acid	-					
Ferulic acid	trans-Ferulic acid	194.0579	$C_{10}H_{10}O_4$	Organic acids	Phenylpropanoids	Cinnamic acids

N-Acetyl-ÃŽÂ±-D-glucosamine	-					
L-Tyrosine	Tyrosine	181.0739	$C_9H_{11}NO_3$	Organic acids	Amino acids and peptides	Amino acids
(8aR,12S,12aR)-12-Hydroxy-4-methyl-4,5,6,7,8,8a,12,12a-octahydro-2H-3-benzoxecine-2,9(1H)-dione	-					
Mannose	Mannose	180.0634	$C_6H_{12}O_6$	Carbohydrates	Monosaccharides	Hexoses
Norepinephrine	Norepinephrine	169.0739	$C_8H_{11}NO_3$	Alkaloids	Tyrosine alkaloids	Phenylethylamines
Ribose	Ribose	150.0528	$C_5H_{10}O_5$	Carbohydrates	Monosaccharides	Pentoses
Benzeneacetic acid	Phenylacetic acid	136.0524	$C_8H_8O_2$	Organic acids	Phenylpropanoids	Cinnamic acids
Succinic acid	Succinic acid	118.0266	$C_4H_6O_4$	Organic acids	TCA acids	TCA acids
Pantothenic acid	Pantothenic acid	219.1107	$C_9H_{17}NO_5$	Organic acids	Amino acids and peptides	Amino acids
Uracil	Uracil	112.0273	$C_4H_4N_2O_2$	Nucleic acids	Pyrimidines	Other pyrimidines
Indole-3-acrylic acid	Indoleacrylic acid	187.0633	$C_{11}H_9NO_2$	Alkaloids	Tryptophan alkaloids	Simple indole alkaloids
2-Hydroxyglutaric acid	2-Hydroxyglutaric acid	148.0372	$C_5H_8O_5$	Fatty Acyls	Fatty acids	Dicarboxylic acids
Trifolin	Kaempferol 3-galactoside	448.1006	$C_{21}H_{20}O_{11}$	Polyketides	Flavonoids	Flavones and Flavonols
Hydroxyproline	Hydroxyproline	131.0582	$C_5H_9NO_3$	Organic acids	Amino acids and peptides	Amino acids
(4S)-4-hydroxy-3,5,5-trimethyl-4-[(1E)-3-{[(2R,3R,4S,5S,6R)-3,4,5-trihydroxy-6-(hydroxymethyl)oxan-2-yl]oxy}but-1-en-1-yl]cyclohex-2-en-1-one	-					
D-(-)-Fructose	Fructose	180.0634	$C_6H_{12}O_6$	Carbohydrates	Monosaccharides	Hexoses

Î±-Hydroxyglutaric acid	2-Hydroxyglutaric acid	148.0372	$C_5H_8O_5$	Fatty Acyls	Fatty acids	Dicarboxylic acids
(2E)-N-(4-acetamidobutyl)-3-(4-hydroxy-3-methoxyphenyl)prop-2-enamide	-					
Cellobiose	Cellobiose	342.1162	$C_{12}H_{22}O_{11}$	Carbohydrates	Disaccharides	Disaccharides
4-Oxoproline	4-Oxo-proline	129.0426	$C_5H_7NO_3$	Organic acids	Amino acids and peptides	Amino acids
4-Hydroxyphenylacetic acid	4-Hydroxyphenylacetic acid	152.0473	$C_8H_8O_3$	Organic acids	Phenylpropanoids	Cinnamic acids
5-hydroxy-4-methoxy-5,6-dihydro-2H-pyran-2-one	5-Hydroxy-4-methoxy-5,6-dihydro-2H-pyran-2-one	144.0423	$C_6H_8O_4$	Organoheterocyclic compounds	Pyranones	Pyranones
Glutaric acid	Glutaric acid	132.0423	$C_5H_8O_4$	Fatty Acyls	Fatty acids	Dicarboxylic acids
Cytidine	Cytidine	243.0855	$C_9H_{13}N_3O_5$	Nucleic acids	Pyrimidines	Pyrimidine ribonucleosides
Lignoceric acid	Lignoceric acid	368.3654	$C_{24}H_{48}O_2$	Fatty Acyls	Fatty acids	Saturated FA
Mannitol	Mannitol	182.079	$C_6H_{14}O_6$	Carbohydrates	Monosaccharides	Sugar alcohols
Benzoic acid	Benzoic acid	122.0368	$C_7H_6O_2$	Benzenoids	Benzenes	Hydroxybenzoic acids
Phenylalanine	Phenylalanine	165.079	$C_9H_{11}NO_2$	Organic acids	Amino acids and peptides	Amino acids
3-[3-(beta-D-Glucopyranosyloxy)-2-hydroxyphenyl]propanoic acid	-					
1-Phenyl-2-butanone	-					
Octopamine	Octopamine	153.079	$C_8H_{11}NO_2$	Alkaloids	Tyrosine alkaloids	Phenylethylamines
Nicotinic acid	Nicotinic acid	123.032	$C_6H_5NO_2$	Alkaloids	Pyridine alkaloids	Nicotinic acid alkaloids

Rutin	Rutin	610.1534	$C_{27}H_{3}0O_{16}$	Polyketides	Flavonoids	Flavones and Flavonols
Quercetin 3-O-rhamnoside-7-O-glucoside	-					
Myoinositol	Myo-inositol	180.0634	$C_6H_{12}O_6$	Organic oxygen compounds	Alcohols and polyols	Inositols
7-hydroxy-6-methoxy-2H-chromen-2-one	Scopoletin	192.0423	$C_{10}H_8O_4$	Polyketides	Flavonoids	Coumarins
Picolinic acid	Picolinic acid	123.032	$C_6H_5NO_2$	Alkaloids	Pyridine alkaloids	Nicotinic acid alkaloids
Betaine	Betaine	117.079	$C_5H_{11}NO_2$	Organic acids	Amino acids and peptides	Amino acids
Caprylic acid	Caprylic acid	144.115	$C_8H_{16}O_2$	Fatty Acyls	Fatty acids	Saturated FA
L-Iditol	L-Iditol	182.079	$C_6H_{14}O_6$	Carbohydrates	Monosaccharides	Sugar alcohols
4-Hydroxybenzoic acid	4-Hydroxybenzoic acid	138.0317	$C_7H_6O_3$	Benzenoids	Benzenes	Hydroxybenzoic acids
Tryptamine	Tryptamine	160.1	$C_{10}H_{12}N_2$	Alkaloids	Tryptophan alkaloids	Tryptamines
Quercitrin	Quercitrin	448.1006	$C_{21}H_{2}0O_{11}$	Polyketides	Flavonoids	Flavones and Flavonols
Shanzhiside methyl ester	-					
N-Acetyltyramine	-					
Vanillic acid	Vanillic acid	168.0423	$C_8H_8O_4$	Benzenoids	Benzenes	Hydroxybenzoic acids
(1S,3R,4R,5R)-1,3,4-trihydroxy-5-{[(2E)-3-(4-hydroxy-3-methoxyphenyl)prop-2-enoyl]oxy}cyclohexane-1-carboxylic acid	-					
Kynurenic acid	Kynurenic acid	189.0426	$C_{10}H_7NO_3$	Alkaloids	Anthranilic acid alkaloids	Acridone alkaloids
2-Amino-2-deoxyhexose	Galactosamine	179.0794	$C_6H_{13}NO_5$	Carbohydrates	Monosaccharides	Amino sugars
Chlorogenic acid	Chlorogenic acid	354.0951	$C_{16}H_{18}O_9$	Organic acids	Phenylpropanoids	Cinnamic acids
Sorbose	Sorbose	180.0634	$C_6H_{12}O_6$	Carbohydrates	Monosaccharides	Hexoses

N-Acetyglucosamine	-					
Guanine	Guanine	151.0494	$C_5H_5N_5O$	Nucleic acids	Purines	Hypoxanthines
Psicofuranose	-					
Isomaltose	Isomaltose	342.1162	$C_{12}H_{22}O_{11}$	Carbohydrates	Disaccharides	Disaccharides
Leucine	Leucine	131.0946	$C_6H_{13}NO_2$	Organic acids	Amino acids and peptides	Amino acids
Glycerol	Glycerol	92.0473	$C_3H_8O_3$	Organic oxygen compounds	Alcohols and polyols	1,2-diols
Gentisic acid	Gentisic acid	154.0266	$C_7H_6O_4$	Benzenoids	Benzenes	Hydroxybenzoic acids
Glucitol	Sorbitol	182.079	$C_6H_{14}O_6$	Carbohydrates	Monosaccharides	Sugar alcohols
Glucopyranose	Glucose	180.0634	$C_6H_{12}O_6$	Carbohydrates	Monosaccharides	Hexoses
Rhamnose	Rhamnose	164.0685	$C_6H_{12}O_5$	Carbohydrates	Monosaccharides	Hexoses
Indole-3-carboxylic acid	Indole-3-carboxylic acid	161.0477	$C_9H_7NO_2$	Alkaloids	Tryptophan alkaloids	Indolecarboxylic acids
Cinnamic acid	trans-Cinnamic acid	148.0524	$C_9H_8O_2$	Organic acids	Phenylpropanoids	Cinnamic acids
(±)9-HpODE	9S-HpODE	312.2301	$C_{18}H_{32}O_4$	Fatty Acyls	Octadecanoids	HpODE
Squalene	Squalene	410.3913	$C_{3}0H_{5}0$	Prenol Lipids	Isoprenoids	C30 isoprenoids
Palmitic acid	Palmitic acid	256.2402	$C_{16}H_{32}O_2$	Fatty Acyls	Fatty acids	Saturated FA
Histidine	Histidine	155.0695	$C_6H_9N_3O_2$	Organic acids	Amino acids and peptides	Amino acids
Protocatechuic acid	Protocatechuic acid	154.0266	$C_7H_6O_4$	Benzenoids	Benzenes	Hydroxybenzoic acids
beta-Sitosterol	Sitosterol	414.3862	$C_{29}H_{5}0O$	Sterol Lipids	Sterols	Stigmasterols
Myristic acid	Myristic acid	228.2089	$C_{14}H_{28}O_2$	Fatty Acyls	Fatty acids	Saturated FA
Stearic acid	Stearic acid	284.2715	$C_{18}H_{36}O_2$	Fatty Acyls	Fatty acids	Saturated FA
8,11,14-Eicosatrienoic acid methyl ester	-					
Ethanolamine	Ethanolamine	61.0528	C_2H_7NO	Organic nitrogen compounds	Amines	1,2-aminoalcohols
Sinapic acid	Sinapic acid	224.0685	$C_{11}H_{12}O_5$	Organic acids	Phenylpropanoids	Cinnamic acids
alpha-Tocopherol	alpha-Tocopherol	430.3811	$C_{29}H_{5}0O_2$	Prenol Lipids	Quinones and hydroquinones	Vitamin E

Methyl linolelaidate	-					
2-Amino-1,3,4-octadecanetriol	-					
13(S)-HOTrE	13S-HOTrE	294.2195	$C_{18}H_{3}0O_{3}$	Fatty Acyls	Octadecanoids	HOTrE
9-Oxo-ODE	9-OxoODE	294.2195	$C_{18}H_{3}0O_{3}$	Fatty Acyls	Octadecanoids	KODE
4-Acetamidobenzoic acid	4-Acetamidobenzoic acid	179.0582	$C_{9}H_{9}NO_{3}$	Benzenoids	Benzenes	Acylaminobenzoic acids
Glutamic acid	Glutamic acid	147.0532	$C_{5}H_{9}NO_{4}$	Organic acids	Amino acids and peptides	Amino acids
Valine	Valine	117.079	$C_{5}H_{11}NO_{2}$	Organic acids	Amino acids and peptides	Amino acids
Tiglylglycine	Tiglylglycine	157.0739	$C_{7}H_{11}NO_{3}$	Organic acids	Amino acids and peptides	Amino acids
Malonic acid	Malonic acid	104.011	$C_{3}H_{4}O_{4}$	Fatty Acyls	Fatty acids	Dicarboxylic acids
Citric acid	Citric acid	192.027	$C_{6}H_{8}O_{7}$	Organic acids	TCA acids	TCA acids
Pyruvic acid	Pyruvic acid	88.016	$C_{3}H_{4}O_{3}$	Organic acids	Short-chain acids	Short-chain acids
Aspartic acid	Aspartic acid	133.0375	$C_{4}H_{7}NO_{4}$	Organic acids	Amino acids and peptides	Amino acids
Benzyl salicylate	Benzyl salicylate	228.0786	$C_{14}H_{12}O_{3}$	Benzenoids	Benzenes	Hydroxybenzoic acids
p-Coumaric acid	cis-p-Coumaric acid	164.0473	$C_{9}H_{8}O_{3}$	Organic acids	Phenylpropanoids	Cinnamic acids
Serine	Serine	105.0426	$C_{3}H_{7}NO_{3}$	Organic acids	Amino acids and peptides	Amino acids
Glutamine	Glutamine	146.0691	$C_{5}H_{10}N_{2}O_{3}$	Organic acids	Amino acids and peptides	Amino acids
2-Aminoadipic acid	alpha-Aminoadipic acid	161.0688	$C_{6}H_{11}NO_{4}$	Organic acids	Amino acids and peptides	Amino acids
Isocitric acid	Isocitric acid	192.027	$C_{6}H_{8}O_{7}$	Organic acids	TCA acids	TCA acids
Gluconic acid	Gluconic acid	196.0583	$C_{6}H_{12}O_{7}$	Carbohydrates	Monosaccharides	Sugar acids
L-Pyroglutamic acid	Pyroglutamic acid	129.0426	$C_{5}H_{7}NO_{3}$	Organic acids	Amino acids and peptides	Amino acids
5-(tert-butyl)-2-methyl-N-(4-nitrophenyl)-3-furamide	-					

2,3,4,9-Tetrahydro-1H -carboline-3-carboxylic acid	-					
Uric acid	Uric acid	168.0283	$C_5H_4N_4O_3$	Nucleic acids	Purines	Xanthines
N-Acetyl-L-leucine	N-Acetylleucine	173.1052	$C_8H_{15}NO_3$	Organic acids	Amino acids and peptides	Amino acids
2,3-Dimethylquinoxaline	-					
Levoglucosan	Glucosan	162.0528	$C_6H_{10}O_5$	Organoheterocyclic compounds	Oxepanes	Oxepanes
D-(+)-Malic acid	D-Malic acid	134.0215	$C_4H_6O_5$	Organic acids	Hydroxy acids	Beta hydroxy acids
Maltose	Maltose	342.1162	$C_{12}H_{22}O_{11}$	Carbohydrates	Disaccharides	Disaccharides
8-{3-Oxo-2-[(2E)-2-penten-1-yl]-1-cyclopenten-1-yl}octanoic acid	-					
6-Hydroxycaproic acid	6-Hydroxycaproic acid	132.0786	$C_6H_{12}O_3$	Fatty Acyls	Fatty acids	Hydroxy FA
Quinoline	Quinoline	129.0578	C_9H_7N	Alkaloids	Anthranilic acid alkaloids	Quinazoline alkaloids
Sucrose	Sucrose	342.1162	$C_{12}H_{22}O_{11}$	Carbohydrates	Disaccharides	Disaccharides
Î±,Î±-Trehalose	-					
D-(+)-Pipecolinic acid	Pipecolic acid	129.079	$C_6H_{11}NO_2$	Organic acids	Amino acids and peptides	Amino acids
Galactaric acid	Galactaric acid	210.0376	$C_6H_{10}O_8$	Fatty Acyls	Fatty acids	Dicarboxylic acids
D-(-)-Quinic acid	Quinic acid	192.0634	$C_7H_{12}O_6$	Benzenoids	Benzenes	Hydroxybenzoic acids
Sucralose	Sucralose	396.0146	$C_{12}H_{19}Cl_3O_8$	Carbohydrates	Disaccharides	Disaccharides
Threonine	Threonine	119.0582	$C_4H_9NO_3$	Organic acids	Amino acids and peptides	Amino acids
Choline	Choline	104.1075	$C_5H_{14}NO$	Organic nitrogen compounds	Cholines	Cholines
Methyl 1,4a-dimethyl-6-methylene-5-[2-(2-oxo-2,5-dihydro-3-	-					

furanyl)ethyl]decahydro-1-naphthalenecarboxylate						
Lysine	Lysine	146.1055	$C_6H_{14}N_2O_2$	Organic acids	Amino acids and peptides	Amino acids
L-Phenylalanine	Phenylalanine	165.079	$C_9H_{11}NO_2$	Organic acids	Amino acids and peptides	Amino acids
Î±-Linolenoyl ethanolamide	alpha-Linolenoyl-EA	321.2668	$C20H_{35}NO_2$	Fatty Acyls	Fatty amides	NAE
2-Deoxyguanosine	Deoxyguanosine	267.0968	$C_{10}H_{13}N_5O_4$	Nucleic acids	Purines	Purine deoxyribonucleosides
Linoleoyl ethanolamide	Linoleoyl-EA	323.2824	$C20H_{37}NO_2$	Fatty Acyls	Fatty amides	NAE
Syringic acid	Syringic acid	198.0528	$C_9H_{10}O_5$	Benzenoids	Benzenes	Hydroxybenzoic acids
Corchorifatty acid F	-					
Vanillin	Vanillin	152.0473	$C_8H_8O_3$	Benzenoids	Benzenes	Hydroxybenzoic acids
Pyrogallol	1,2,3-Trihydroxybenzene	126.0317	$C_6H_6O_3$	Benzenoids	Phenols	Benzenetriols
Nicotinamide	Niacinamide	122.048	$C_6H_6N_2O$	Alkaloids	Pyridine alkaloids	Nicotinic acid alkaloids
Adenine	Adenine	135.0545	$C_5H_5N_5$	Nucleic acids	Purines	Aminopurines
Vanillylmandelic acid	Vanillylmandelic acid	198.0528	$C_9H_{10}O_5$	Organic acids	Phenylpropanoids	Cinnamic acids
Tryptophan	Tryptophan	204.0899	$C_{11}H_{12}N_2O_2$	Organic acids	Amino acids and peptides	Amino acids
Cystine	Cystine	240.0239	$C_6H_{12}N_2O_4S_2$	Organic acids	Amino acids and peptides	Amino acids
9S,13R-12-Oxophytodienoic acid	-					
Cholesterol	Cholesterol	386.3549	$C_{27}H_{46}O$	Sterol Lipids	Sterols	Cholesterols
2-Hydroxybutanedioic acid	-					

Table S2. Tentatively identified metabolites using UHPLC-ESI-MS (+/-) data.

Name	Formula	Annot. DeltaMass [ppm]	*m/z*	RT [min]	mzCloud Best Match	Adduct
Choline	$C_5 H_{13} N O$	-0.91	104.1069	0.698	98.9	[M+H]+1
Adenine	$C_5 H_5 N_5$	-1.42	136.0616	0.749	53.5	[M+H]+1
Norepinephrine	$C_8 H_{11} N O_3$	-0.1	170.0812	0.757	93.4	[M+H]+1
L-Iditol	$C_6 H_{14} O_6$	-1.37	181.0715	0.783	95	[M-H]-1
D-(-)-Fructose	$C_6 H_{12} O_6$	0.22	179.0562	0.796	83	[M-H]-1
α,α-Trehalose	$C_{12} H_{22} O_{11}$	-0.89	341.1086	0.832	98.5	[M-H]-1
Gluconic acid	$C_6 H_{12} O_7$	-0.25	195.051	0.841	53	[M-H]-1
Guanine	$C_5 H_5 N_5 O$	-0.03	152.0567	0.871	78.2	[M+H]+1
D-(-)-Quinic acid	$C_7 H_{12} O_6$	-0.61	191.056	0.872	50.7	[M-H]-1
N-Acetyl-α-D-glucosamine	$C_8 H_{15} N O_6$	0.15	222.0972	0.879	99.8	[M+H]+1
5-hydroxy-4-methoxy-5,6-dihydro-2H-pyran-2-one	$C_6 H_8 O_4$	0.05	145.0495	0.886	78.7	[M+H]+1
D-(+)-Pipecolinic acid	$C_6 H_{11} N O_2$	0.36	130.0863	0.9	96.4	[M+H]+1
Picolinic acid	$C_6 H_5 N O_2$	0.17	124.0393	0.906	69.1	[M+H]+1
Glutaric acid	$C_5 H_8 O_4$	0.84	131.0351	0.908	85.7	[M-H]-1
L-Pyroglutamic acid	$C_5 H_7 N O_3$	0.3	130.0499	0.922	98.9	[M+H]+1
N-Acetyl-L-leucine	$C_8 H_{15} N O_3$	-0.09	174.1125	0.928	65.1	[M+H]+1
α-Hydroxyglutaric acid isomer 1	$C_5 H_8 O_5$	0.73	147.03	0.935	53.7	[M-H]-1
Nicotinamide	$C_6 H_6 N_2 O$	-0.72	123.0552	0.937	58.8	[M+H]+1
3-[3-(beta-D-Glucopyranosyloxy)-2-hydroxyphenyl]propanoic acid	$C_{15} H_{20} O_9$	-0.66	343.1032	0.947	95.8	[M-H]-1
Betaine	$C_5 H_{11} N O_2$	-1.4	118.0861	0.959	99.5	[M+H]+1
L-Tyrosine	$C_9 H_{11} N O_3$	0.1	182.0812	0.965	99	[M+H]+1
D-(+)-Malic acid	$C_4 H_6 O_5$	0.06	133.0143	0.987	99.6	[M-H]-1
4-Oxoproline isomer 1	$C_5 H_7 N O_3$	1.17	128.0355	0.989	100	[M-H]-1
Apocynin	$C_9 H_{10} O_3$	-0.04	167.0703	0.99	60.3	[M+H]+1

α-Hydroxyglutaric acid isomer 2	$C_5H_8O_5$	1.53	147.0301	1.266	58.4	[M-H]-1
L-Phenylalanine	$C_9H_{11}NO_2$	-0.09	166.0862	1.333	99.7	[M+H]+1
4-Oxoproline isomer 2	$C_5H_7NO_3$	1.87	128.0356	1.454	99.9	[M-H]-1
Isocitric acid	$C_6H_8O_7$	1.03	191.0199	1.579	90.6	[M-H]-1
Shanzhiside methyl ester	$C_{17}H_{26}O_{11}$	0.17	405.1403	3.136	63.4	[M-H]-1
N-Acetyltyramine	$C_{10}H_{13}NO_2$	-0.05	180.1019	4.06	99.5	[M+H]+1
2,3,4,9-Tetrahydro-1H-β-carboline-3-carboxylic acid	$C_{12}H_{12}N_2O_2$	0.39	217.0972	4.555	52.3	[M+H]+1
Chlorogenic acid	$C_{16}H_{18}O_9$	-0.93	353.0875	4.625	99	[M-H]-1
7-hydroxy-6-methoxy-2H-chromen-2-one isomer 1	$C_{10}H_8O_4$	-0.04	193.0495	4.813	96.7	[M+H]+1
6-Hydroxycaproic acid	$C_6H_{12}O_3$	0.55	131.0714	5.092	84.8	[M-H]-1
(1S,3R,4R,5R)-1,3,4-trihydroxy-5-{[(2E)-3-(4-hydroxy-3-methoxyphenyl)prop-2-enoyl]oxy}cyclohexane-1-carboxylic acid	$C_{17}H_{20}O_9$	-0.445	367.1033	5.17	98.4	[M-H]-1
(4S)-4-hydroxy-3,5,5-trimethyl-4-[(1E)-3-{[(2R,3R,4S,5S,6R)-3,4,5-trihydroxy-6-(hydroxymethyl)oxan-2-yl]oxy}but-1-en-1-yl]cyclohex-2-en-1-one	$C_{19}H_{30}O_8$	0.26	387.2015	5.2	95.4	[M+H]+1
Rutin	$C_{27}H_{30}O_{16}$	1.54	609.1471	5.307	97.2	[M-H]-1
1-Phenyl-2-butanone	$C_{10}H_{12}O$	-0.02667	149.0961	5.413667	78.4	[M+H]+1
Quercetin	$C_{15}H_{10}O_7$	0.32	303.05	5.509	97.1	[M+H]+1
D(+)-Phenyllactic acid	$C_9H_{10}O_3$	0.23	165.0558	5.552	97.7	[M-H]-1
(2E)-N-(4-acetamidobutyl)-3-(4-hydroxy-3-methoxyphenyl)prop-2-enamide	$C_{16}H_{22}N_2O_4$	0.26	307.1653	5.5775	51.6	[M+H]+1
Quercitrin	$C_{21}H_{20}O_{11}$	0.34	449.108	5.745	99.7	[M+H]+1

Quercetin-3β-D-glucoside isomer 1	$C_{21}H_{20}O_{12}$	0.11	465.1028	5.75	99.9	[M+H]+1
1,3,5-trihydroxy-4-{[(2E)-3-(3-hydroxy-4-methoxyphenyl)prop-2-enoyl]oxy}cyclohexane-1-carboxylic acid	$C_{17}H_{20}O_{9}$	0.4	369.1182	5.783	96.7	[M+H]+1
4-Acetamidobenzoic acid	$C_{9}H_{9}NO_{3}$	-0.09	178.051	5.862	75.4	[M-H]-1
7-hydroxy-6-methoxy-2H-chromen-2-one isomer 2	$C_{10}H_{8}O_{4}$	-0.13	193.0495	5.935	99.2	[M+H]+1
Trifolin	$C_{21}H_{20}O_{11}$	0.45	449.108	5.978	99.5	[M+H]+1
Kaempferol	$C_{15}H_{10}O_{6}$	0.455	287.0551	5.988	93.6	[M+H]+1
Isorhamnetin	$C_{16}H_{12}O_{7}$	0.43	317.0657	6.018	95.5	[M+H]+1
Kynurenic acid	$C_{10}H_{7}NO_{3}$	-0.06	190.0499	6.102	97.6	[M+H]+1
Quinoline	$C_{9}H_{7}N$	0.46	130.0652	6.168	94.6	[M+H]+1
2,3-Dimethylquinoxaline	$C_{10}H_{10}N_{2}$	0.06	159.0917	6.169	96.1	[M+H]+1
Indole-3-acrylic acid	$C_{11}H_{9}NO_{2}$	0.03	188.0706	6.169	95.3	[M+H]+1
Quercetin-3β-D-glucoside isomer 2	$C_{21}H_{20}O_{12}$	0.38	465.1029	6.189	96.5	[M+H]+1
Quercetin 3-O-rhamnoside-7-O-glucoside	$C_{27}H_{30}O_{16}$	0.37	611.1609	6.191	98.1	[M+H]+1
(8aR,12S,12aR)-12-Hydroxy-4-methyl-4,5,6,7,8,8a,12,12a-octahydro-2H-3-benzoxecine-2,9(1H)-dione	$C_{14}H_{20}O_{4}$	0.16	253.1435	6.21	70.3	[M+H]+1
Corchorifatty acid F	$C_{18}H_{32}O_{5}$	-0.685	327.2175	6.805	60.5	[M-H]-1
8-{3-Oxo-2-[(2E)-2-penten-1-yl]-1-cyclopenten-1-yl}octanoic acid	$C_{18}H_{28}O_{3}$	0.36	293.2112	6.868	79.8	[M+H]+1
Methyl 1,4a-dimethyl-6-methylene-5-[2-(2-oxo-2,5-dihydro-3-furanyl)ethyl]decahydro-1-naphthalenecarboxylate	$C_{21}H_{30}O_{4}$	0.41	347.2218	6.934	85	[M+H]+1

5-(tert-butyl)-2-methyl-N-(4-nitrophenyl)-3-furamide	$C_{16}H_{18}N_2O_4$	0.45	303.1341	6.957	65.8	[M+H]+1
Ferulic acid	$C_{10}H_{10}O_4$	0.02	195.0652	7.157	86.1	[M+H]+1
9S,13R-12-Oxophytodienoic acid isomer 2	$C_{18}H_{28}O_3$	0.32	275.2006	7.361	93.6	[M+H-H2O]+1
9S,13R-12-Oxophytodienoic acid isomer 1	$C_{18}H_{28}O_3$	0.29	293.2112	7.662	75.4	[M+H]+1
2-Amino-1,3,4-octadecanetriol	$C_{18}H_{39}NO_3$	0.39	318.3004	7.983	95.2	[M+H]+1
13(S)-HOTrE isomer 1	$C_{18}H_{30}O_3$	-0.67	293.212	8.66	75.1	[M-H]-1
Gentisic acid	$C_7H_6O_4$	0.56	153.0194	8.671	85.7	[M-H]-1
13(S)-HOTrE isomer 2	$C_{18}H_{30}O_3$	-0.62	293.212	9.224	96.7	[M-H]-1
(±)9-HpODE	$C_{18}H_{32}O_4$	-0.62	311.2226	9.232	91.3	[M-H]-1
13(S)-HOTrE isomer 3	$C_{18}H_{30}O_3$	-0.55	293.2121	9.363	56.8	[M-H]-1
α-Linolenoyl ethanolamide	$C_{20}H_{35}NO_2$	0.55	322.2742	10.55	96.3	[M+H]+1
9-Oxo-ODE isomer 2	$C_{18}H_{30}O_3$	0.44	277.2163	10.63	98.8	[M+H-H2O]+1
9-Oxo-ODE isomer 1	$C_{18}H_{30}O_3$	0.43	277.2163	10.741	98.5	[M+H-H2O]+1
Linoleoyl ethanolamide	$C_{20}H_{37}NO_2$	0.45	324.2899	11.206	97.2	[M+H]+1

Table S3. Tentatively identified TMS-derivatized metabolites using GC-MS data. SI, Similarity Index; RI, retention index.

Name	RT [min]	NIST Lib Hit Formula	SI	Calculated RI	RI Delta
(E)-Cinnamic acid, 1TMS	11.49	$C_{12}H_{16}O_2Si$	669	1550	3
2-Amino-2-deoxyhexose, 5TMS	15.806	$C_{21}H_{53}NO_5Si_5$	786	1950	52
2-Aminoadipic acid, 3TMS	13.341	$C_{15}H_{35}NO_4Si_3$	610	1712	41
2-Deoxyguanosine, 4TMS	22.614	$C_{22}H_{45}N_5O_4Si_4$	797	2778	42
2-Hydroxybutanedioic acid, 3TMS	10.324	$C_{13}H_{30}O_5Si_3$	812	1455	19
2-Hydroxyglutaric acid, 3TMS	11.78	$C_{14}H_{32}O_5Si_3$	846	1574	39
4-Hydroxy-3-methoxyphenylglycol, 3TMS	16.291	$C_{18}H_{36}O_4Si_3$	880	2002	162
4-Hydroxybenzoic acid, 2TMS	12.362	$C_{13}H_{22}O_3Si_2$	802	1625	51

4-Hydroxyphenylacetic acid, 2TMS	12.474	$C_{14} H_{24} O_3 Si_2$	665	1635	17
8,11,14-Eicosatrienoic acid methyl ester	18.213	$C_{21} H_{36} O_2$	775	2214	25
alpha-Tocopherol, 1TMS	24.995	$C_{32} H_{58} O_2 Si$	911	3133	14
Benzeneacetic acid, 1TMS	8.265	$C_{11} H_{16} O_2 Si$	788	1297	2
Benzoic acid, 1TMS	7.5355	$C_{10} H_{14} O_2 Si$	691	1241	4
Benzyl salicylate	15.73	$C_{14} H_{12} O_3$	611	1942	73
beta-Sitosterol, 1TMS	26.249	$C_{32} H_{58} O Si$	893	3344	20
Caffeic acid, 3TMS	17.5	$C_{18} H_{32} O_4 Si_3$	916	2133	17
Caprylic acid, 1TMS	8.67	$C_{11} H_{24} O_2 Si$	704	1321	87
Chlorogenic acid, 6TMS	25.112	$C_{34} H_{66} O_9 Si_6$	888	3106	3
Cholesterol, 1TMS	25.067	$C_{30} H_{54} O Si$	840	3145	20
Citric acid, 4TMS	14.441	$C_{18} H_{40} O_7 Si_4$	838	1814	32
Cytidine, 4TMS	21.971	$C_{21} H_{45} N_3 O_5 Si_4$	697	2687	184
D-(+)-Cellobiose isomer 1, 8TMS	21.832	$C_{36} H_{86} O_{11} Si_8$	655	2668	35
D-(+)-Mannose, 1MOX, 5TMS	15.2965	$C_{22} H_{55} N O_6 Si_5$	856	1887	4
D-(+)-Xylose, 1MOX, 4TMS	13.0345	$C_{18} H_{45} N O_5 Si_4$	781	1676	34
D-Gluconic acid, 6TMS	16.352	$C_{24} H_{60} O_7 Si_6$	701	2009	105
D-Gluconolactone, 4TMS	15.085	$C_{18} H_{42} O_6 Si_4$	775	1876	7
DL-Phenylalanine, 1TMS	11.367	$C_{12} H_{19} N O_2 Si$	824	1533	9
DL-Proline, 2TMS	8.204	$C_{11} H_{25} N O_2 Si_2$	815	1292	22
DL-Serine, 3TMS	9.067	$C_{12} H_{31} N O_3 Si_3$	802	1357	2
DL-Threonine, 3TMS	9.399	$C_{13} H_{33} N O_3 Si_3$	839	1382	8
D-Mannitol, 6TMS	17.426	$C_{24} H_{62} O_6 Si_6$	814	2118	163
D-Pantothenic acid, 3TMS	16.134	$C_{18} H_{41} N O_5 Si_3$	816	1985	87
D-Psicofuranose isomer 1, 5TMS	13.944	$C_{21} H_{52} O_6 Si_5$	686	1769	67
Ethanolamine, 3TMS	7.287	$C_{11} H_{31} N O Si_3$	847	1226	4
Ferulic acid isomer 1, 2TMS	17.139	$C_{16} H_{26} O_4 Si_2$	859	2092	160
Galactaric acid, (R,S,R,S)-, 6TMS	16.312	$C_{24} H_{58} O_8 Si_6$	688	2004	58
Glucitol, 6TMS	15.563	$C_{24} H_{62} O_6 Si_6$	861	1924	14
Glucopyranose, 5TMS	16.059	$C_{21} H_{52} O_6 Si_5$	855	1977	46
Glucose, 5TMS	16.524	$C_{21} H_{52} O_6 Si_5$	717	2027	40
Glutamic acid, 3TMS	12.273	$C_{14} H_{33} N O_4 Si_3$	863	1617	1
Glyceric acid, 3TMS	8.663	$C_{12} H_{30} O_4 Si_3$	833	1326	17
Glycerol, 3TMS	7.943	$C_{12} H_{32} O_3 Si_3$	820	1273	16
Guanine, 3TMS	17.4	$C_{14} H_{29} N_5 O Si_3$	947	2122	13
Indole-3-carboxylic acid, 2TMS	16.465	$C_{15} H_{23} N O_2 Si_2$	739	2021	10
Isomaltose isomer 1, 1MOX, 8TMS	22.271	$C_{37} H_{89} N O_{11} Si_8$	765	2729	102
Isomaltose isomer 2, 8TMS	21.809	$C_{36} H_{86} O_{11} Si_8$	723	2665	182
Kynurenic acid, 2TMS	16.872	$C_{16} H_{23} N O_3 Si_2$	783	2064	22
L-Aspartic acid, 3TMS	11.139	$C_{13} H_{31} N O_4 Si_3$	800	1520	78
Leucine, 2TMS	7.7015	$C_{12} H_{29} N O_2 Si_2$	872	1269	6

Levoglucosan, 3TMS	14.366	$C_{15} H_{34} O_5 Si_3$	750	1808	159
L-Glutamine, 3TMS	13.985	$C_{14} H_{34} N_2 O_3 Si_3$	881	1772	4
L-Histidine, 3TMS	15.485	$C_{15} H_{33} N_3 O_2 Si_3$	726	1915	3
L-Hydroxyproline, (E)-, 2TMS	13.079	$C_{11} H_{25} N O_3 Si_2$	718	1688	190
Lignoceric acid, 1TMS	22.643	$C_{27} H_{56} O_2 Si$	803	2732	85
L-Tryptophan, 3TMS	18.144	$C_{20} H_{36} N_2 O_2 Si_3$	677	2206	97
L-Valine, 2TMS	6.949	$C_{11} H_{27} N O_2 Si_2$	924	1202	10
Lysine, 4TMS	15.489	$C_{18} H_{46} N_2 O_2 Si_4$	811	1916	17
Malonic acid, 2TMS	7.4	$C_9 H_{20} O_4 Si_2$	621	1234	48
Maltose isomer 1, 8TMS	20.395	$C_{36} H_{86} O_{11} Si_8$	657	2479	152
Methyl linolelaidate	18.147	$C_{19} H_{34} O_2$	762	2207	96
Myoinositol, 6TMS	17.14	$C_{24} H_{60} O_6 Si_6$	762	2092	6
Myristic acid, 1TMS	14.747	$C_{17} H_{36} O_2 Si$	865	1844	21
N-Acetyl-D-glucosamine isomer 1, 1MOX, 4TMS	16.917	$C_{21} H_{50} N_2 O_6 Si_4$	613	2069	93
N-Acetyl-D-glucosamine isomer 2, 1MOX, 4TMS	16.995	$C_{21} H_{50} N_2 O_6 Si_4$	758	2077	77
Nicotinic acid, 1TMS	8.1575	$C_9 H_{13} N O_2 Si$	795	1285	5
Octopamine, 3TMS	15.55	$C_{17} H_{35} N O_2 Si_3$	690	1923	186
Palmitic acid, 1TMS	16.6865	$C_{19} H_{40} O_2 Si$	834	2044	13
p-Coumaric acid, 2TMS	15.6935	$C_{15} H_{24} O_3 Si_2$	875	1933	13
Protocatechuic acid, 3TMS	14.422	$C_{16} H_{30} O_4 Si_3$	859	1813	16
Pyrogallol, 3TMS	12.035	$C_{15} H_{30} O_3 Si_3$	632	1596	78
Pyruvic acid, 2TMS	7.383	$C_9 H_{20} O_3 Si_2$	788	1233	134
Quercetin, 5TMS	25.252	$C_{30} H_{50} O_7 Si_5$	745	3177	43
Rhamnose isomer 1, 4TMS	13.067	$C_{18} H_{44} O_5 Si_4$	686	1687	77
Ribose isomer 1, 4TMS	10.993	$C_{17} H_{42} O_5 Si_4$	656	1508	105
Ribose isomer 2, 4TMS	13.821	$C_{17} H_{42} O_5 Si_4$	660	1757	136
Ribose isomer 2, 4TMS	12.589	$C_{17} H_{42} O_5 Si_4$	645	1645	32
Sinapic acid, 2TMS	18.443	$C_{17} H_{28} O_5 Si_2$	797	2242	33
Sorbose isomer 1, 1MOX, 5TMS	15.058	$C_{22} H_{55} N O_6 Si_5$	872	1873	2
Squalene	22.845	$C_{30} H_{50}$	826	2811	0
Stearic acid, 1TMS	18.4445	$C_{21} H_{44} O_2 Si$	844	2242	15
Succinic acid, 2TMS	8.2515	$C_{10} H_{22} O_4 Si_2$	854	1312	5
Sucralose, 5TMS	19.542	$C_{27} H_{59} Cl_3 O_8 Si_5$	621	2372	157
Sucrose, 8TMS	21.531	$C_{36} H_{86} O_{11} Si_8$	855	2627	3
Syringic acid, 2TMS	15.22	$C_{15} H_{26} O_5 Si_2$	874	1889	6
Tiglylglycine, 1TMS	11.06	$C_{10} H_{19} N O_3 Si$	606	1514	63
Tryptamine, 2TMS	16.52133	$C_{19} H_{36} N_2 Si_3$	775	2031	88
Tryptamine, 3TMS	18.723	$C_{19} H_{36} N_2 Si_3$	633	2273	154
Tyrosine, 3TMS	15.704	$C_{18} H_{35} N O_3 Si_3$	807	1939	10
Uracil, 2TMS	8.635667	$C_{10} H_{20} N_2 O_2 Si_2$	845	1316	3
Uric acid, 4TMS	17.171	$C_{17} H_{36} N_4 O_3 Si_4$	689	2096	97

Vanillic acid, 2TMS	13.864	$C_{14} H_{24} O_4 Si_2$	828	1761	1
Vanillin, 1MOX, 1TMS	12.607	$C_{12} H_{19} N O_3 Si$	620	1647	14
Vanillylmandelic acid, 3TMS	14.567	$C_{18} H_{34} O_5 Si_3$	757	1827	58

Table S4. Venn analysis results displaying the total unique and common tentative metabolites between the extracts, as shown in Figure 6.

Extracts	Total	Features
LSA LSE LSM LSW	77	Quercetin-3beta-D-glucoside
		Xylose
		Kaempferol
		Gluconolactone
		Tyrosine
		4-Hydroxy-3-methoxyphenylglycol
		Apocynin
		D(+)-Phenyllactic acid
		Isorhamnetin
		Caffeic acid
		Glyceric acid
		Glucose
		Quercetin
		Proline
		1,3,5-trihydroxy-4-{[(2E)-3-(3-hydroxy-4-methoxyphenyl)prop-2-enoyl]oxy}cyclohexane-1-carboxylic acid
		Ferulic acid
		N-Acetyl-D-glucosamine
		L-Tyrosine
		(8aR,12S,12aR)-12-Hydroxy-4-methyl-4,5,6,7,8,8a,12,12a-octahydro-2H-3-benzoxecine-2,9(1H)-dione
		Mannose
		Norepinephrine
		Ribose
		Benzeneacetic acid
		Succinic acid
		Pantothenic acid
		Uracil

Indole-3-acrylic acid
2-Hydroxyglutaric acid
Trifolin
Hydroxyproline
(4S)-4-hydroxy-3,5,5-trimethyl-4-[(1E)-3-{[(2R,3R,4S,5S,6R)-3,4,5-trihydroxy-6-(hydroxymethyl)oxan-2-yl]oxy}but-1-en-1-yl]cyclohex-2-en-1-one
D-(-)-Fructose
α-Hydroxyglutaric acid
(2E)-N-(4-acetamidobutyl)-3-(4-hydroxy-3-methoxyphenyl)prop-2-enamide
Cellobiose
4-Oxoproline
4-Hydroxyphenylacetic acid
5-hydroxy-4-methoxy-5,6-dihydro-2H-pyran-2-one
Glutaric acid
Cytidine
Lignoceric acid
Mannitol
Benzoic acid
Phenylalanine
3-[3-(beta-D-Glucopyranosyloxy)-2-hydroxyphenyl]propanoic acid
1-Phenyl-2-butanone
Octopamine
Nicotinic acid
Rutin
Quercetin 3-O-rhamnoside-7-O-glucoside
Myoinositol
7-hydroxy-6-methoxy-2H-chromen-2-one
Picolinic acid
Betaine
Caprylic acid
L-Iditol
4-Hydroxybenzoic acid
Tryptamine
Quercitrin
Shanzhiside methyl ester

		N-Acetyltyramine
		Vanillic acid
		(1S,3R,4R,5R)-1,3,4-trihydroxy-5-{[(2E)-3-(4-hydroxy-3-methoxyphenyl)prop-2-enoyl]oxy}cyclohexane-1-carboxylic acid
		Kynurenic acid
		2-Amino-2-deoxyhexose
		Chlorogenic acid
		Sorbose
		N-Acetyglucosamine
		Guanine
		Psicofuranose
		Isomaltose
		Leucine
		Glycerol
		Gentisic acid
		Glucitol
		Glucopyranose
		Rhamnose
LSA LSE LSM	19	Indole-3-carboxylic acid
		Cinnamic acid
		(±)9-HpODE
		Squalene
		Palmitic acid
		Histidine
		Protocatechuic acid
		beta-Sitosterol
		Myristic acid
		Stearic acid
		8,11,14-Eicosatrienoic acid methyl ester
		Ethanolamine
		Sinapic acid
		alpha-Tocopherol
		Methyl linolelaidate
		2-Amino-1,3,4-octadecanetriol
		13(S)-HOTrE

		9-Oxo-ODE
		4-Acetamidobenzoic acid
LSE LSM LSW	13	Glutamic acid
		Valine
		Tiglylglycine
		Malonic acid
		Citric acid
		Pyruvic acid
		Aspartic acid
		Benzyl salicylate
		p-Coumaric acid
		Serine
		Glutamine
		2-Aminoadipic acid
		Isocitric acid
LSA LSM LSW	24	Gluconic acid
		L-Pyroglutamic acid
		5-(tert-butyl)-2-methyl-N-(4-nitrophenyl)-3-furamide
		2,3,4,9-Tetrahydro-1H-carboline-3-carboxylic acid
		Uric acid
		N-Acetyl-L-leucine
		2,3-Dimethylquinoxaline
		Levoglucosan
		D-(+)-Malic acid
		Maltose
		8-{3-Oxo-2-[(2E)-2-penten-1-yl]-1-cyclopenten-1-yl}octanoic acid
		6-Hydroxycaproic acid
		Quinoline
		Sucrose
		alpha, alpha-Trehalose
		D-(+)-Pipecolinic acid
		Galactaric acid
		D-(-)-Quinic acid
		Sucralose
		Threonine

		Choline
		Methyl 1,4a-dimethyl-6-methylene-5-[2-(2-oxo-2,5-dihydro-3-furanyl)ethyl]decahydro-1-naphthalenecarboxylate
		Lysine
		L-Phenylalanine
LSA LSE LSW	10	Alpha-Linolenoyl ethanolamide
		2-Deoxyguanosine
		Linoleoyl ethanolamide
		Syringic acid
		Corchorifatty acid F
		Vanillin
		Pyrogallol
		Nicotinamide
		Adenine
		Vanillylmandelic acid
LSE LSM	1	Tryptophan
LSM LSW	1	Cystine
LSA LSW	1	9S,13R-12-Oxophytodienoic acid
LSA	2	Cholesterol
		2-Hydroxybutanedioic acid

Table S5. Venn analysis results displaying unique and common tentatively identified phytochemicals between extracts, as shown in Figure 8.

Names	total	elements
LSA LSE LSM LSW	28	Rutin
		Quercetin-3beta-D-glucoside
		Benzeneacetic acid
		Kaempferol
		Quercetin 3-O-rhamnoside-7-O-glucoside
		4-Hydroxy-3-methoxyphenylglycol
		Indole-3-acrylic acid
		7-hydroxy-6-methoxy-2H-chromen-2-one
		Apocynin
		Trifolin
		Picolinic acid
		D(+)-Phenyllactic acid
		Vanillic acid

		Kynurenic acid
		Isorhamnetin
		Chlorogenic acid
		4-Hydroxyphenylacetic acid
		Caffeic acid
		Quercetin
		4-Hydroxybenzoic acid
		Ferulic acid
		Benzoic acid
		Tryptamine
		Gentisic acid
		Quercitrin
		Octopamine
		Norepinephrine
		Nicotinic acid
LSA LSE LSM	7	Protocatechuic acid
		Indole-3-carboxylic acid
		Sinapic acid
		Cinnamic acid
		alpha-Tocopherol
		Squalene
		4-Acetamidobenzoic acid
LSE LSM LSW	2	Benzyl salicylate
		p-Coumaric acid
LSA LSM LSW	2	D-(-)-Quinic acid
		Quinoline
LSA LSE LSW	4	Pyrogallol
		Vanillin
		Syringic acid
		Vanillylmandelic acid
LSA LSE	1	Nicotinamide
LSE	1	beta-Sitosterol

Table S6. ANOVA analysis (p<0.05) showing the significant tentative phytochemicals that contribute to the separation among extracts, as shown in Figure S6.

(A) GC-MS

**Phytochemical name**	**F.stat**	***p*-value**	**-log10(p)**	**FDR**
p-Coumaric acid	436.66	3.33E-09	8.4773	8.66E-08
alpha-Tocopherol	343.14	8.68E-09	8.0617	1.13E-07

Protocatechuic acid	128.04	4.23E-07	6.3737	3.67E-06
4-Hydroxybenzoic acid	102.24	1.02E-06	5.9927	6.61E-06
Vanillylmandelic acid	80.197	2.61E-06	5.5841	1.35E-05
beta-Sitosterol	75.645	3.26E-06	5.4863	1.40E-05
4-Hydroxy-3-methoxyphenylglycol	72.897	3.76E-06	5.4245	1.40E-05
4-Hydroxyphenylacetic acid	62.923	6.61E-06	5.1796	2.15E-05
Vanillic acid	58.588	8.68E-06	5.0613	2.51E-05
Nicotinic acid	55.57	1.06E-05	4.9739	2.76E-05
Squalene	48.867	1.73E-05	4.7626	4.08E-05
Cinnamic acid	42.181	3.00E-05	4.5226	6.50E-05
Chlorogenic acid	37.909	4.47E-05	4.3498	8.94E-05
Caffeic acid	32.086	8.27E-05	4.0828	0.000153
Pyrogallol	24.478	0.00022	3.6576	0.000381
Benzoic acid	22.558	0.000294	3.5316	0.000452
Benzyl salicylate	22.52	0.000296	3.5289	0.000452
Octopamine	21.673	0.000339	3.4703	0.000489
Indole-3-carboxylic acid	18.312	0.000609	3.2154	0.000833
Quercetin	9.8847	0.004567	2.3403	0.005938
Tryptamine	9.5843	0.005019	2.2994	0.006215
Kynurenic acid	9.1495	0.005777	2.2383	0.006828
Benzeneacetic acid	8.0301	0.008502	2.0705	0.009611
Syringic acid	5.4113	0.025055	1.6011	0.027143
Vanillin	4.1344	0.048137	1.3175	0.050062

(B) UHPLC-ESI-MS (+/-)

**Phytochemical name**	**F.stat**	***p*-value**	**-log10(p)**	**FDR**
Kynurenic acid	271.94	2.18E-08	7.6615	4.80E-07
Quercetin 3-O-rhamnoside-7-O-glucoside	139.98	2.98E-07	6.5254	3.28E-06

D-(-)-Quinic acid	112.25	7.07E-07	6.1506	5.18E-06
Quinoline	97.407	1.23E-06	5.911	6.75E-06
Indole-3-acrylic acid	89.907	1.67E-06	5.776	7.37E-06
Quercitrin	78.192	2.87E-06	5.5417	1.05E-05
Rutin	68.023	4.91E-06	5.3091	1.41E-05
Quercetin-3Î²-D-glucoside	67.268	5.12E-06	5.2905	1.41E-05
7-hydroxy-6-methoxy-2H-chromen-2-one	52.316	1.34E-05	4.8745	3.26E-05
4-Acetamidobenzoic acid	48.555	1.77E-05	4.7521	3.89E-05
Apocynin	41.75	3.12E-05	4.5059	6.24E-05
Isorhamnetin	34.077	6.63E-05	4.1788	0.000121
Quercetin	33.372	7.16E-05	4.1454	0.000121
Ferulic acid	30.928	9.45E-05	4.0244	0.000149
Trifolin	27.054	0.000154	3.8134	0.000225
Nicotinamide	15.827	0.001001	2.9998	0.001313
Chlorogenic acid	15.761	0.001015	2.9937	0.001313
D(+)-Phenyllactic acid	14.604	0.001309	2.8829	0.0016
Kaempferol	12.417	0.002228	2.6522	0.002579
Norepinephrine	11.598	0.002771	2.5573	0.003048
Gentisic acid	4.7096	0.035412	1.4509	0.037098
